# Regulation of epithelial migration by epithelial cell adhesion molecule requires its Claudin-7 interaction domain

**DOI:** 10.1371/journal.pone.0204957

**Published:** 2018-10-10

**Authors:** Angela I. M. Barth, Honesty Kim, Ingmar H. Riedel-Kruse

**Affiliations:** Department of Bioengineering, Stanford University, Stanford, CA, United States of America; NCMLS, Radboud University Nijmegen Medical Center, NETHERLANDS

## Abstract

Epithelial cell adhesion molecule (EpCAM) is a glycoprotein on the surface of epithelial cells that is essential for intestinal epithelial integrity and expressed at high levels in many epithelial derived cancers and circulating tumor cells. Here we show the effect of EpCAM levels on migration of Madin-Darby-Canine Kidney (MDCK) epithelial cells. MDCK cells depleted of EpCAM show increased activation of extracellular signal-regulated kinase (ERK) and of myosin, and increased cell spreading and epithelial sheet migration into a gap. In contrast, over-expression of EpCAM inhibits ERK and myosin activation, and slows epithelial sheet migration. Loss of EpCAM is rescued by EpCAM-YFP mutated in the extracellular domain required for cis-dimerization whereas EpCAM-YFP with a mutation that inhibits Claudin-7 interaction cannot rescue increased ERK, myosin activation, and increased migration in EpCAM-depleted cells. In summary, these results indicate that interaction of EpCAM and Claudin-7 at the cell surface negatively regulates epithelial migration by inhibiting ERK and actomyosin contractility.

## Introduction

Epithelial cell adhesion molecule (EpCAM) is a glycoprotein on the surface of epithelial cells that has been described to mediate cell-cell adhesion by homophilic interaction [1–4]. The structure of the extracellular domain of EpCAM shows a cis-dimer that may interact in trans with another EpCAM cis-dimer on a neighboring cell surface [5]. Although EpCAM dimers have been detected in cell lysates, only a small portion of cellular EpCAM seems to be dimerized [6]. Compared to a strong epithelial cell adhesion molecule such as E-cadherin, homophilic adhesion mediated by EpCAM is weak [1,2]. Even so, EpCAM is essential for intestinal epithelial integrity in mice and humans [7–10]. In humans, mutations in EpCAM cause congenital tufting enteropathy (CTE), a disease which is associated with loss of tight junction protein Claudin-7 and increased actomyosin contractility at epithelial cell-cell junctions leading to the disruption of intestinal epithelial integrity [9–11]. EpCAM has key signaling functions during embryonic development [12–14] and is expressed at high levels in epithelial derived cancers and circulating tumor cells [15–19]. However, its roles in epithelial homoeostasis and cancer progression are poorly understood.

EpCAM may contribute indirectly to epithelial adhesion by regulating other adhesion systems [2,8,13,20,21]. Notably, EpCAM interacts with tight junction protein Claudin-7 and stabilizes Claudin-7 at the surface of epithelial cells [6,22–24]. Loss of EpCAM leads to loss of Claudin-7 from intestinal epithelial cells in mice and to defective tight junctions [8,24]. During *Xenopus laevis* embryonic development, loss of EpCAM causes increased actomyosin contractility which destabilizes C-cadherin at cell-cell adhesion sites [13].

In *X*. *laevis* embryos, EpCAM regulates actomyosin contractility by inhibiting novel protein kinase C (nPKC) activity at the membrane, thereby attenuating activation of extracellular signal-regulated kinase ERK and phosphorylation of non-muscle myosin regulatory light chain [13,25]. EpCAM cytoplasmic domain is required for inhibition of nPKC but its extracellular domain is not, indicating that EpCAM-mediated adhesion is not important for this regulation [13,25]. In epithelial sheets of human enterocytes, loss of EpCAM results in increased actomyosin contractile forces at tricellular contacts which causes pathological tuft formation in CTE [11].

Actomyosin contractility is required for epithelial sheet migration [26]. Recent evidence suggests that EpCAM attenuates ERK signaling in mammalian epithelial cells, thereby slowing epithelial migration, but EpCAM-dependent changes in phospho-myosin levels in cortical F-actin were not investigated during migration [27]. Since epithelial cell-cell junctions are linked to cortical F-actin [28], and Claudin-7 localizes to tight junctions while also being distributed along the basolateral membrane of epithelial cells [29], the question arises whether EpCAM interaction with Claudin-7 is required to inhibit epithelial migration. Here we investigated whether EpCAM regulates phospho-myosin levels in cortical F-actin during epithelial migration and whether EpCAM interaction with Claudin-7 is required for EpCAM’s function in signaling and epithelial migration.

## Results

### EpCAM regulates epithelial sheet migration

**Fig 1:**
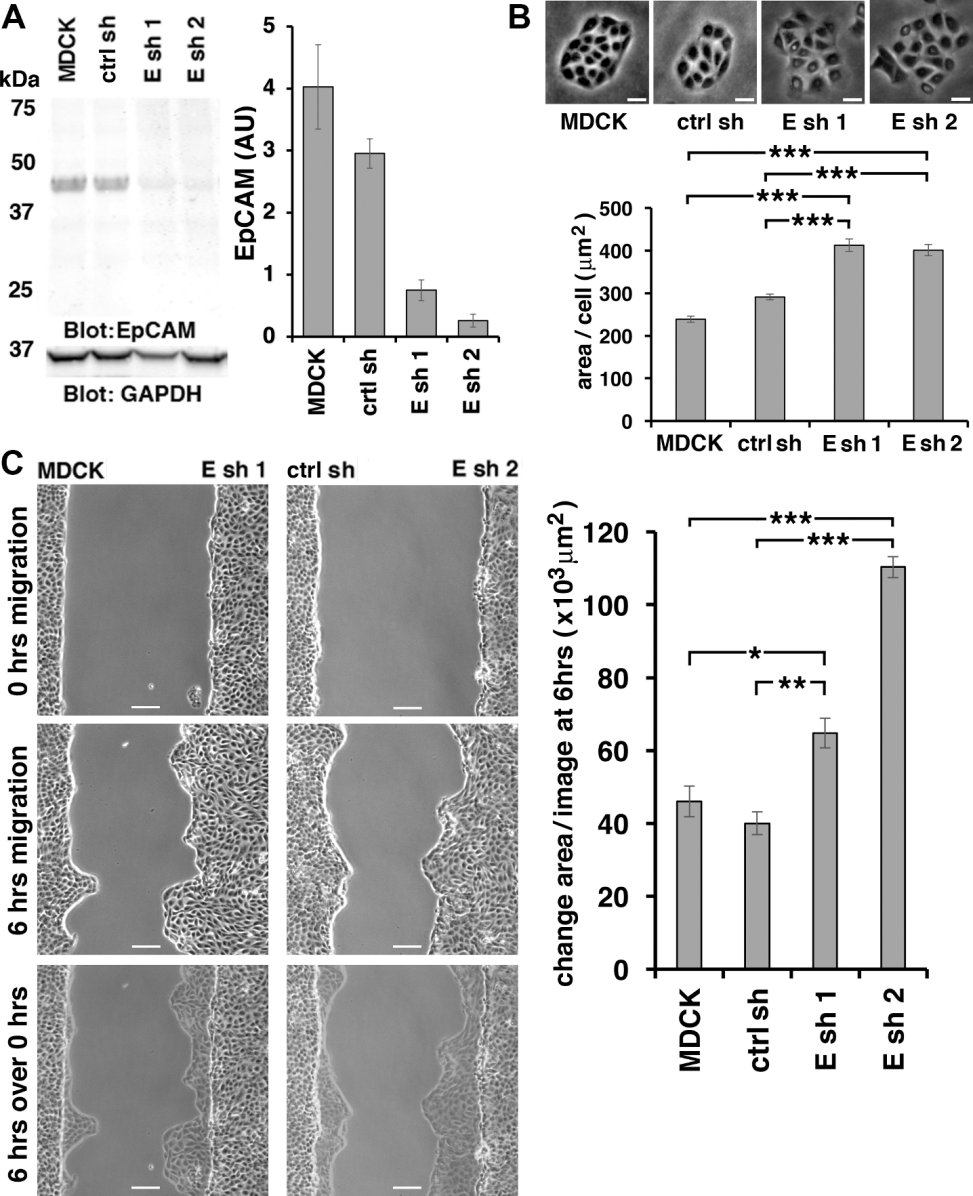
EpCAM-depletion promotes cell spreading and epithelial sheet migration. **(A)** SDS extracts from MDCK cells or MDCK cell lines expressing either control shRNA (ctrl sh) or anti-EpCAM shRNA 1 or 2 (Esh1; Esh2) were immunoblotted for EpCAM and GAPDH. Graph shows quantification of EpCAM protein levels normalized to GAPDH protein levels in the same samples. Error bars: S.E.M. of three samples for each cell line. Compared to parental MDCK cells, EpCAM was 5x reduced in Esh1 and 15x reduced in Esh2 expressing cells; compared to ctrl sh EpCAM was 4x reduced in Esh1 and 11x reduced in Esh2 expressing cells. (**B)** Phase images (top) and quantification of area/cell (graph) of individual colonies of indicated cell lines. Scale bars: 20 μm. Area per cell was significantly increased in EpCAM depleted cell lines Esh1 and Esh2 with ***p values < 0.0001 (two-tailed Mann-Whitney) compared to MDCK or ctrl sh lines. Error bars: S.E.M. of 90 MDCK, 88 ctrl sh, 104 Esh1 and 58 Esh2, small colonies of 5 to 200 cells **(C)** Phase images of indicated cell lines at 0 hrs (top row) and at 6 hrs migration (middle row). The bottom row shows an overlay of “6 hrs” images onto “0 hrs” images. Scale bars: 100 μm. Graph in (C) shows quantification of average change of area/image after 6 hrs migration for each cell line. Error bars: S.E.M. of nine (MDCK), four (ctrl sh, Esh1) and three (Esh2) experiments. Stars correspond to *p* values derived from an unpaired Student’s *t*-test. For Esh1 *p =* 0.034 compared to MDCK and *p =* 0.003 compared to ctrl sh; and for Esh2 *p*< 0.001 compared to MDCK or ctrl sh.

MDCK epithelial cells depleted of EpCAM with two different small hairpin (sh) RNAs (Esh1 and Esh2, Fig 1A) show increased cell spreading on substrate compared to parental MDCK cells or MDCK cells expressing a control shRNA (Fig 1B). This morphology is consistent with a more migratory phenotype [30]. Accordingly, EpCAM-depleted epithelial sheets migrate faster into a gap (Fig 1C, S1 and S2 Movies). A similar effect of EpCAM loss of function on migration has been observed in other epithelial cells [27].

**Fig 2:**
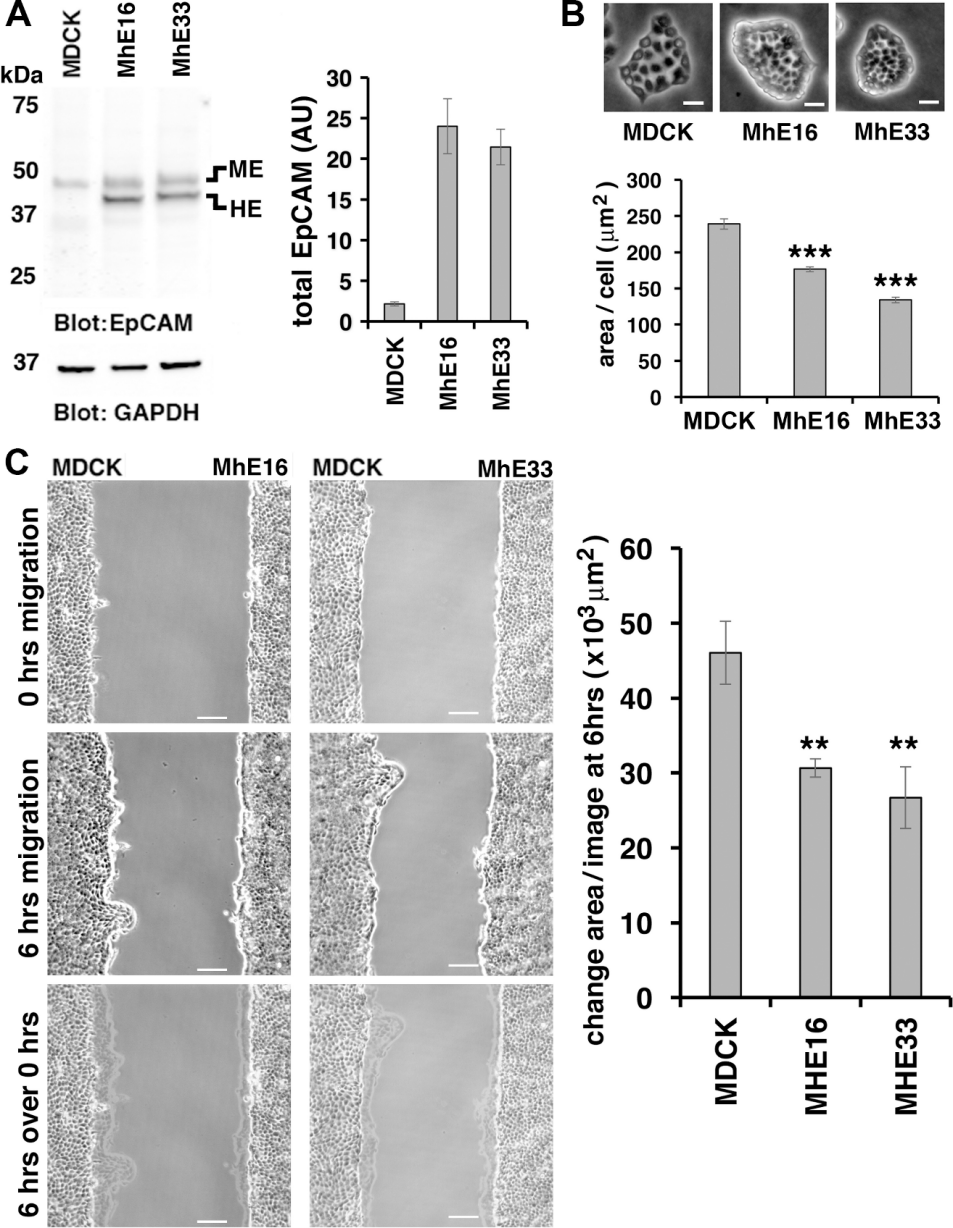
EpCAM-overexpression inhibits cell spreading and epithelial sheet migration. **(A)** SDS extracts from MDCK cells Mhe16 and Mhe33 lines were immunoblotted for EpCAM and GAPDH. MDCK cells express endogenous EpCAM (ME) whereas MhE lines express both ME and untagged human EpCAM (HE). Graph shows quantification of EpCAM protein levels normalized to GAPDH protein levels in the same samples. For total EpCAM protein levels in Mhe16 and MhE33, intensities for MDCK-endogenous EpCAM (ME) and human EpCAM (HE) were combined. Error bars: S.E.M. of three samples for each cell line. Compared to parental MDCK cells, the EpCAM level was 11x higher in MhE16 and 10x higher in MhE33 cells. (**B)** Phase images (top) and quantification of area/cell (graph) of individual colonies of indicated cell lines. Scale bars: 20 μm. Area per cell was significantly decreased in MhE16 and MhE33 cells with ***p values < 0.0001 (two-tailed Mann-Whitney) compared to MDCK cells. Error bars: S.E.M. of 90 MDCK, 268 MhE16 and 284 MhE33 small colonies of 5 to 200 cells. (**C)** Phase images of indicated cell lines at 0 hrs migration (top row) and at 6 hrs migration (middle row). The bottom row shows an overlay of “6 hrs” images onto “0 hrs” images. Scale bars: 100 μm. Graph in (C) shows quantification of average change of area/image after 6 hrs migration for each cell line. Bars: S.E.M. of nine (MDCK), four (MhE16) or five (MhE33) experiments. MDCK values are the same as shown in the graph of Fig 1C. Stars correspond to *p* values derived from unpaired Student’s *t*-test. For MhE16 **p = 0.0063 and for MhE33 **p = 0.0074 compared to MDCK.

To test whether increased levels of EpCAM would have the opposite effects on morphology and migration, we expressed human EpCAM in MDCK cells (Fig 2A). Two independent MDCK cell lines expressing human EpCAM (MhE16 and MhE33) show decreased cell spreading on substrate compared to parental MDCK cells (Fig 2B). Epithelial sheets with increased levels of EpCAM migrate slower into a gap than parental MDCK cells (Fig 2C, S3 and S4 Movies).

### EpCAM reduces baseline phospho-ERK activity in growing cultures and ERK activation in response to growth factors

**Fig 3:**
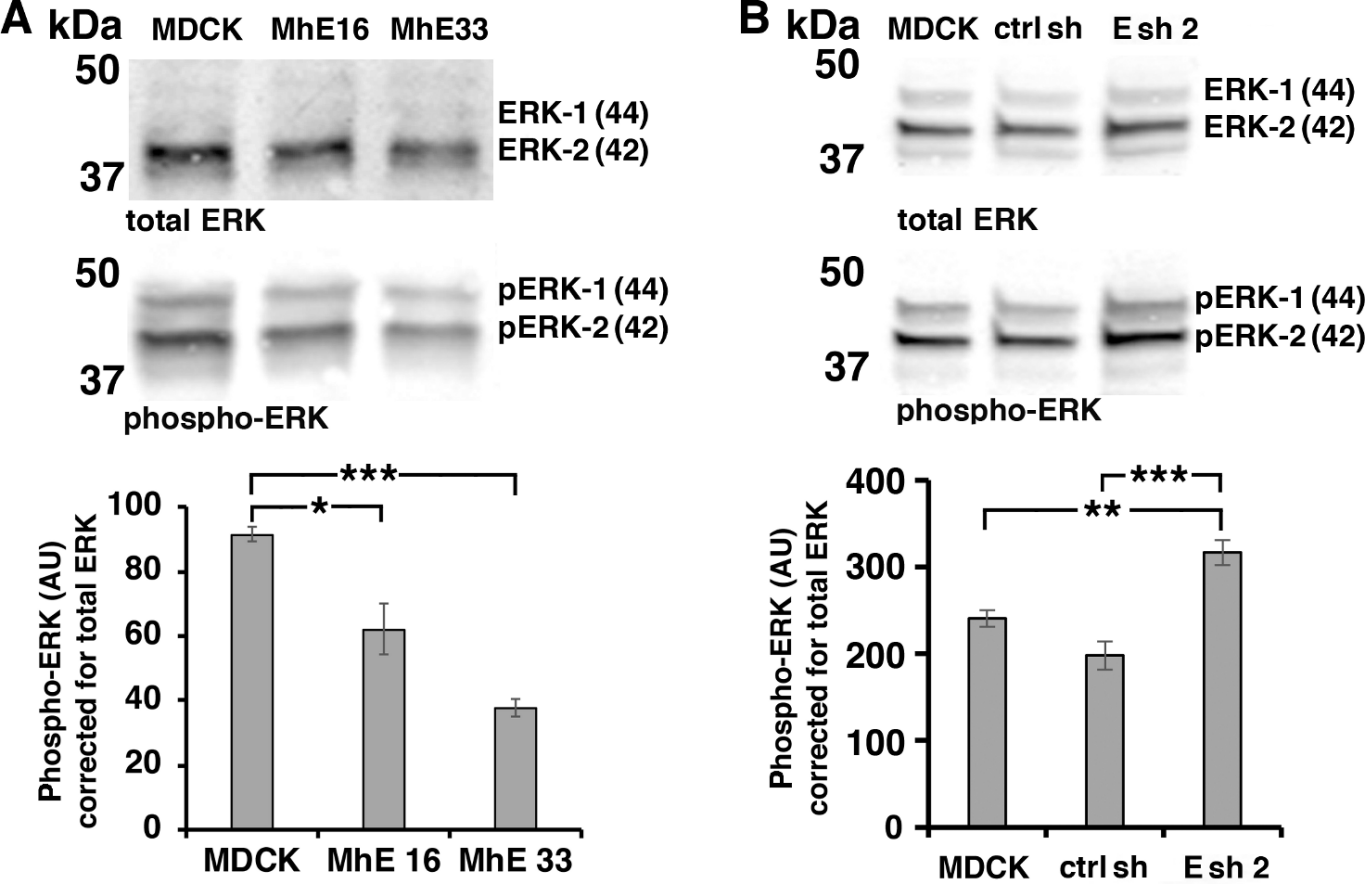
EpCAM inhibits ERK activation. **(A)** Wildtype MDCK, and MDCK lines overexpressing human EpCAM (MhE16, MhE33) were SDS extracted one day after plating at subconfluent cell density; levels of ERK and phospho-ERK were analyzed in the same immunoblot. The graph shows quantification of combined 44 and 42 kDa phospho-ERK protein levels normalized to combined 44 and 42 kDa ERK protein levels in the same sample. Error bars: S.E.M. of three independent samples for each cell line; *p* values derived from unpaired Student’s *t* test: * *p* = 0.023 for MhE16 to MDCK and *** *p* = 0.0001 for MhE33 to MDCK. **(B)** Phospho-ERK levels in MDCK cells, control shRNA-expressing MDCK cells (ctrl sh) and EpCAM-depleted MDCK cells (Esh2) were analyzed as in (A). Error bars: S.E.M. of six independent samples for each cell line; *p* values derived from unpaired Student’s *t* test: ** *p* = 0.0013 for Esh2 to MDCK and *** *p* = 0.0003 for Esh2 to ctrl sh.

Activity of extracellular-signal regulated kinase (ERK) promotes epithelial migration [31,32]. EpCAM has been shown to negatively regulate ERK activity during *Xenopus laevis* embryonic development [13] and in mammalian epithelial cells [27]. Indeed, baseline levels of phospho-ERK activity in growing cultures are significantly decreased in MDCK lines MhE16 and MhE33 overexpressing EpCAM (Fig 3A) whereas ERK activity is significantly increased in MDCK cell line Esh2 depleted of EpCAM (Fig 3B) compared to parental MDCK cells or a cell line expressing control shRNA (ctrl sh). ERK activity is upregulated by hepatocyte growth factor (HGF)/scatter factor which stimulates MDCK migration [30]. To test whether EpCAM inhibits ERK activation in response to HGF, parental MDCK cells and MhE lines overexpressing EpCAM were serum-starved for two hours (S1 Fig) and treated with HGF in the absence of serum for the indicated time intervals (S1C–S1F Fig). ERK activity in response to HGF is significantly decreased in MhE16 and MhE33 lines compared to parental MDCK (S1C–S1E Fig). Since MhE16 and MhE33 have already strongly decreased baseline levels of phospho-ERK before HGF treatment (S1A and S1B Fig), we determined fold activation over 0 HGF (S1F Fig). The increase in activated ERK compared to the baseline level in serum-starved cells without HGF is not significantly different between the EpCAM-overexpressing lines and parental MDCK cells (S1F Fig).

### EpCAM regulates phospho-myosin accumulation in cortical F-actin and formation of phospho-myosin-rich leader cells

**Fig 4:**
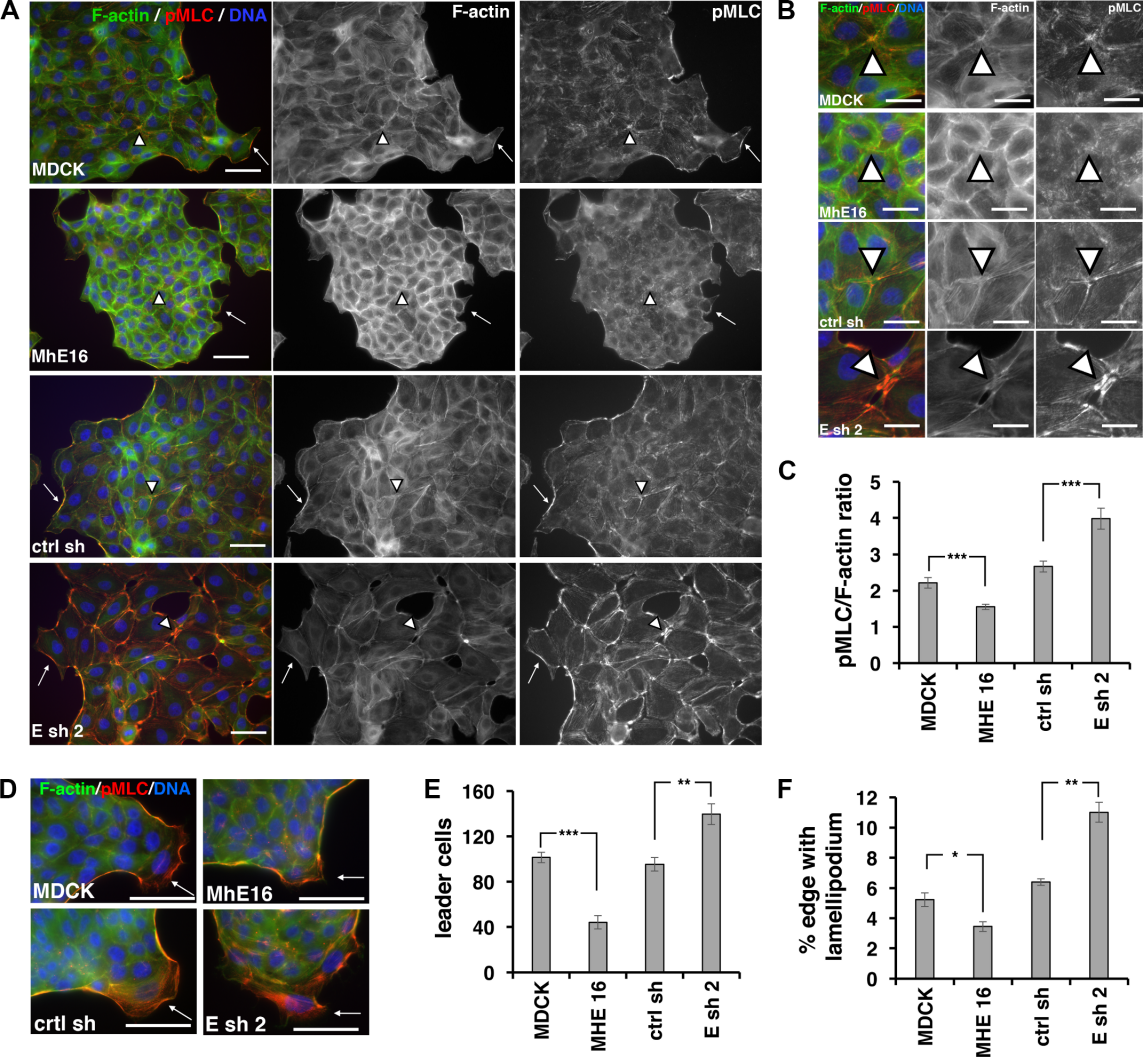
EpCAM regulates phospho-myosin levels along cortical F-actin and formation of phospho-myosin-rich leader cells. **(A)** Low-density, two-day cell cultures on collagen stained for F-actin (green), phospho-myosin (pMLC, red) and DNA (blue), and single channel images for F-actin, and for phospho-myosin (pMLC). Phospho-myosin-rich areas of cortical F-actin at the edge of colonies are marked with arrows and phospho-myosin-rich multicellular junctions inside colonies are marked with arrowheads. Bars = 50 μm. **(B)** Magnification of images in (A) showing the phospho-myosin rich junctions marked with arrowheads. Bars = 25 μm. **(C)** Phospho-myosin (pMLC) / F-actin ratios in cortical F-actin areas were determined for each cell line shown in (A, B) with at least 30 images of 0.087mm^2^. Error bars = S.E.M.; ***p < 0.0001 for MDCK and MhE16; ***p = 0.000114 for ctrl sh and Esh2 in two-tailed Mann-Whitney test. **(D)** Merged images showing edges of epithelial sheets with leader cells (arrows) at 6 hours migration after well removal stained for F-actin (green), phospho-myosin (pMLC, red) and DNA (blue). Bars = 50 μm. **(E)** Average number of leader cells formed at 6 hours migration in a monolayer with a starting area of 0.35 cm^2^. Error bars = S.E.M. of four experiments; ***p < 0.0001 for MDCK and MhE16 and **p = 0.005 for ctrl sh and Esh2 in unpaired Student’s *t* test. **(F)** Percent cell sheet edge with lamellipodium. Error bars = S.E.M. of four experiments; ***p < 0.0213 for MDCK and MhE16; ***p = 0.0037 for ctrl sh and Esh2 in unpaired Student’s *t* test.

ERK activity promotes phosphorylation of non-muscle myosin regulatory light chain (MLC) [13,33] which is required for myosin localization to F-actin and actomyosin contractility [34,35]. In *Xenopus laevis* embryos loss of EpCAM function leads to increased ERK activity and increased levels of phospho-myosin [13]. To test whether EpCAM decreases phospho-myosin levels in MDCK cells, we analyzed phospho-myosin accumulation in cortical F-actin by immunofluorescence (Fig 4). The level of phospho-myosin in cortical F-actin is increased in low density cultures of MDCK cells depleted of EpCAM (Esh2 in Fig 4A–4C) and decreased in MDCK cells overexpressing EpCAM (MhE16 and MhE33 in Fig 4A–4C; examples of smaller colonies are given in S2 Fig).

Compared to control MDCK cell colonies, EpCAM-overexpressing MhE16 and MhE33 cell are compact (Fig 4A and 4B; see also Fig 2B), have less phospho-myosin in cortical F-actin at the colony’s edge (arrows in Fig 4A and 4B) and show less enrichment of phospho-myosin in multicellular junctions inside of the colonies (arrowheads in Fig 4A and 4B). The combined phospho-myosin/F-actin ratio of cortical areas between cells and at the colony edge is significantly reduced in the EpCAM-overexpressing cell line MhE16 compared to parental MDCK cells (Fig 4C).

Compared to control MDCK and ctrl-shRNA-expressing cell colonies, EpCAM-depleted Esh2 cell colonies are spread out and their cortical F-actin is enriched for phospho-myosin (arrows in Fig 4A and 4B; phospho-myosin /F-actin ratio in cortical F-actin in Fig 4C, see also Fig 1B and S1 Fig). Multicellular junctions inside EpCAM-depleted colonies are especially enriched for phospho-myosin compared to multicellular junctions inside colonies of control lines or EpCAM-overexpressing lines (arrowheads in Fig 4A and 4B). EpCAM-depleted cultures have gaps at phospho-myosin-rich multicellular junctions (arrowheads in Fig 4B: Esh2), indicating loss of cell-cell adhesion caused by increased actomyosin contractility.

In migration assays with high cell density epithelial sheets, leader cells provide cues to the monolayer leading it into a gap (arrows in Fig 4D) [36,37]. The number of phospho-myosin-rich leader cells formed at 6 hours of migration is strongly reduced in EpCAM-overexpressing cells and increased in EpCAM-depleted cells (Fig 4E). The combined length of all lamellipodia, including leader cell lamellipodia (arrows in Fig 4D) compared to total length of the epithelial sheet border is reduced in EpCAM-overexpressing lines and increased in EpCAM-depleted lines (Fig 4F).

### EpCAM regulates Claudin-7 levels and localization in kidney epithelial cells

**Fig 5:**
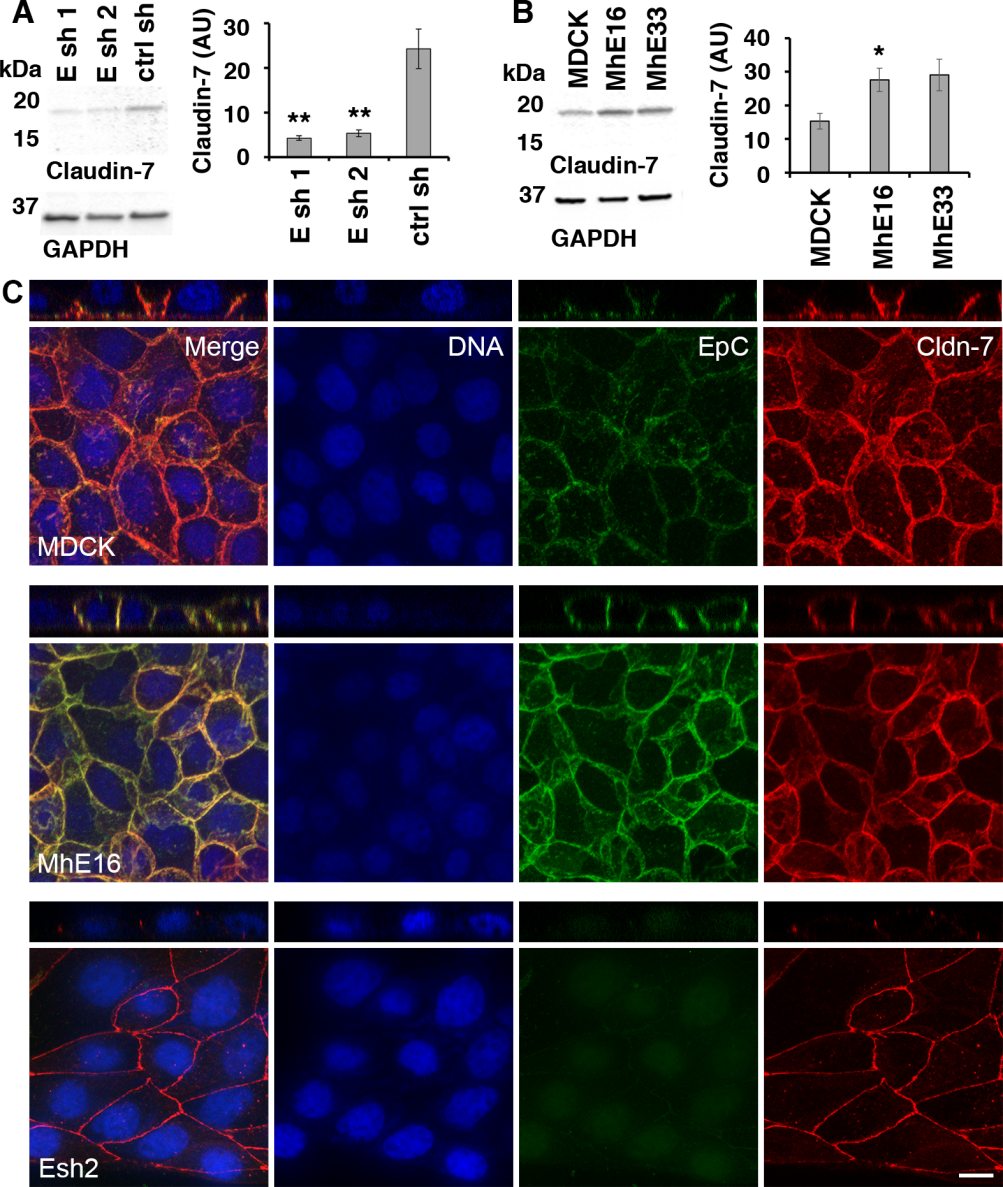
Levels and localization of tight junction protein Claudin-7 are regulated by EpCAM in MDCK cells. **(A)** EpCAM-depleted MDCK lines Esh1, Esh2 and control shRNA line ctrl sh were SDS extracted one day after plating at subconfluent cell density. Protein levels of Claudin-7 and of GAPDH as a loading control were analyzed in the same immunoblot. The graphs show quantification of Claudin-7 protein normalized to GAPDH levels in the same sample. Error bars: S.E.M. of four samples for each cell line; *p* values derived from unpaired Student’s *t* test: ** *p* = 0.0042 for Esh1 to ctrl sh and ** *p* = 0.0057 for Esh2 to ctrl sh. **(B)** Claudin-7 protein levels in MDCK cells and cell lines MhE16, MhE33 overexpressing human EpCAM were analyzed as described in (A). Arbitrary units for protein intensities in Y-axis (AU) x10^3^; error bars: S.E.M. of three samples for each cell line; *p* value derived from unpaired Student’s *t* test: * *p* = 0.042 for MhE16 to MDCK. **(C)** Confocal images were acquired of cells 24 hours after plating on IBIDI chambers and 0 hours after removal of the stencil. The figure shows maximum intensity projections and orthogonal projections of confocal stacks of MDCK, MhE16 and Esh2 cells stained for nuclei (blue), EpCAM (green), and Claudin-7 (red). In the MDCK and MhE16 lines, both EpCAM and Claudin-7 localize along basolateral membrane. In the Esh2 line, Claudin-7 does not distribute throughout the basolateral membrane, and EpCAM staining is very weak. In Esh2 line, greater contrast was applied post-processing for clarity. Nuclear signal in EpCAM channel in Esh2 is due to residual mCherry signal in the nucleus post methanol fixation. Scale bar is 10μm.

Loss of EpCAM causes loss of Claudin-7 from the intestinal epithelium which is associated with congenital tufting enteropathy and loss of intestinal epithelial integrity in mice and humans [8,9]. Therefore, we analyzed whether Claudin-7 protein levels are regulated by EpCAM protein in MDCK epithelial cells (Fig 5). In EpCAM-depleted MDCK cells Claudin-7 levels are significantly reduced to one-fifth of their level in control shRNA cells (Fig 5A) and in MDCK cells overexpressing EpCAM Claudin-7 protein levels are slightly increased to approximately twice the amount compared to parental MDCK cells (Fig 5B).

Confocal imaging of EpCAM shows localization to the basolateral membrane (Fig 5C: MDCK and MhE16). For the EpCAM overexpressing line, there is also a small amount of EpCAM present on the apical surface of the membrane (Fig 5C: MhE16 z-projection). Both wild-type and overexpressing lines show colocalization of Claudin-7 with EpCAM to the basolateral membrane. However in EpCAM-depleted MDCK lines, Claudin-7 no longer localizes along the basolateral membrane (Fig 5C: Esh2), and instead is restricted to the tight-junctions (S3 and S4 Figs).

Loss of EpCAM also causes reductions in the levels of other Claudins, such Claudin-1–2, -3, -7 and -15 [8,24]. Interaction of these other Claudins with EpCAM may be indirect, as has been shown for the association of Claudin-1 with EpCAM which requires Claudin-7 [24]. In MDCK cells depleted of EpCAM, we observed only a slight reduction in Claudin-1 protein level (S5A Fig) whereas Claudin-3 protein level is significantly reduced (S5B Fig). To analyze whether other junctional proteins are affected, we tested E-cadherin protein levels in EpCAM-depleted and EpCAM-overexpressing MDCK cell lines (S6 Fig). Overall E-cadherin protein levels were only slightly affected but, in contrast to Claudins, E-cadherin levels were slightly increased in EpCAM-depleted MDCK cells (S6A Fig) and slightly decreased in EpCAM-overexpressing MDCK cells (S6B Fig). In summary, these results indicate that EpCAM is required to maintain Claudin levels but is not required to maintain E-cadherin levels in MDCK cells.

### Rescue of Claudin-7 levels and localization in EpCAM-depleted cells by expression of EpCAM-YFP mutant proteins

**Fig 6:**
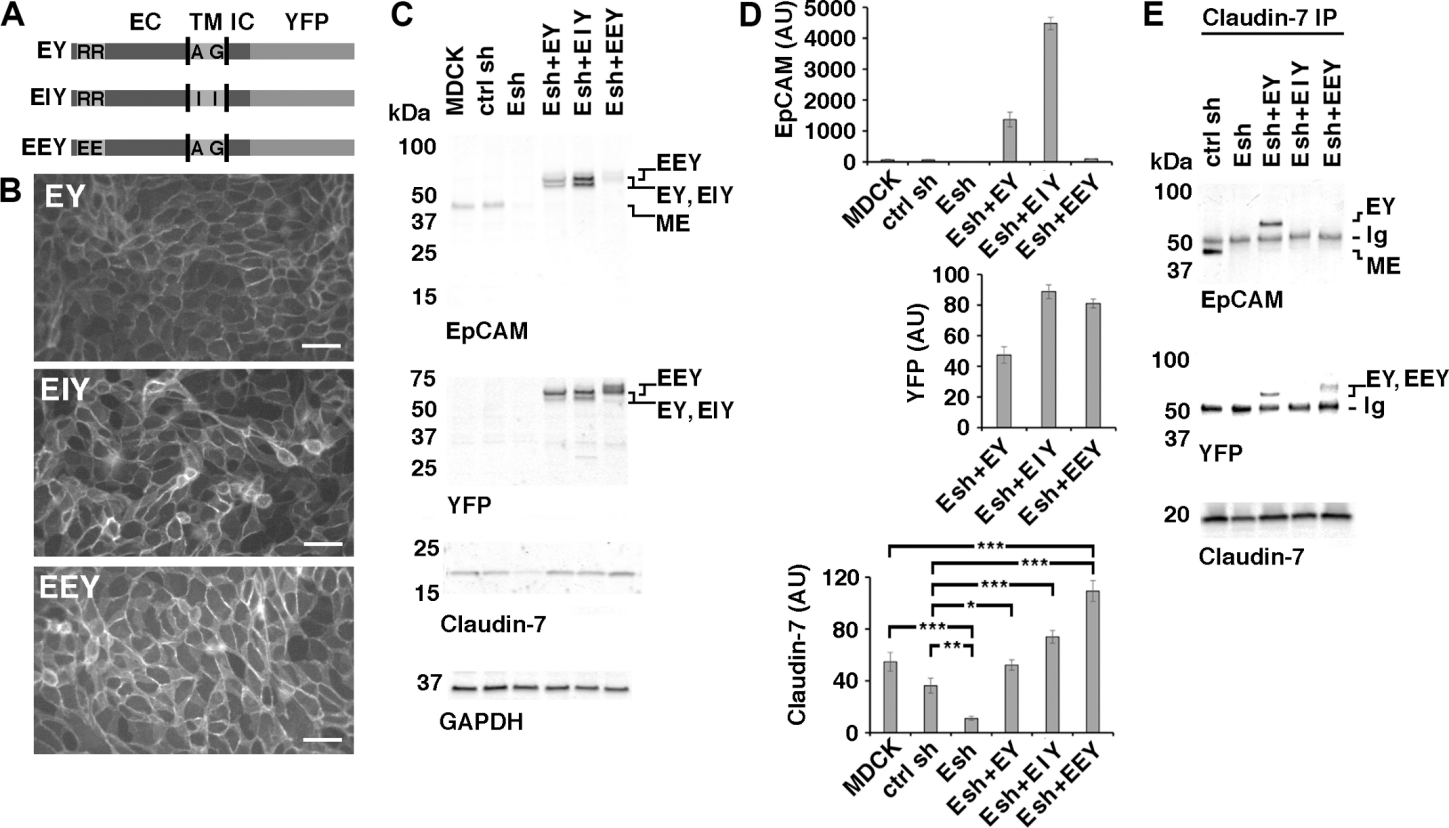
Rescue of Claudin-7 protein levels by expression of EpCAM-YFP or point mutants of EpCAM-YFP in MDCK cells depleted of EpCAM. **(A)** Schematic of EpCAM-YFP (EY) and EpCAM-YFP mutant proteins (EIY and EEY). Extracellular domain (EC), transmembrane domain (TM), intracellular domain (IC) and YFP fusion are indicated. Approximate position of amino acids mutated in EIY and EEY are indicated with their one letter codes. **(B)** EpCAM-depleted Esh2-derived cell lines expressing EY, EIY or EEY were plated at confluent density onto an IBIDI 4 well dish for one day and YFP fluorescence was imaged before well chambers were removed for a migration assay. YFP fluorescence localizes to cell-cell contacts in all three cell lines. Scale bars: 50 μm. **(C)** Indicated MDCK cell lines were cultured, extracted and protein levels were analyzed as described for Figs 1A, 2A and 5. EpCAM-YFP fusion proteins EY, EIY and EEY appear as double bands; MDCK-endogenous EpCAM (ME) is a single band. EEY appears slightly larger and is poorly recognized by the EpCAM extracellular domain antibody (top EpCAM blot). Expression of Claudin-7 is reduced in the parental Esh2 line (Esh) compared to MDCK and control shRNA line ctrl sh (Claudin-7 blot; see also Fig 5). **(D)** Graphs show quantifications of indicated proteins normalized to GAPDH levels in the same sample. For EpCAM protein levels, residual MDCK-endogenous EpCAM (ME) and EpCAM-YFP protein double band levels (EY, EIY, EEY) were combined to show total EpCAM levels in the Esh2 lines expressing EY, EIY or EEY. Total EpCAM is ~ 20x or ~21x higher in Esh + EY and ~ 64x or ~68x higher in Esh + EIY compared to MDCK or ctrl sh cell lines. Please note that the EpCAM antibody was made against human EpCAM and may have different affinities for the endogenous canine EpCAM in MDCK and ctrl sh cell lines and the exogenous human EpCAM-YFP proteins, so these values are only approximations. Since EEY is poorly recognized by EpCAM antibody, expression levels of the three YFP fusion proteins are best compared in the YFP immunoblot (middle YFP blot). Error bars: S.E.M. of six samples for each cell line. Expression of EY, EIY or EEY in Esh 2 rescues Claudin-7 protein levels in these cell lines compared to Claudin-7 level in the Esh 2 line (Esh in Claudin-7 graph): *p* values are derived from unpaired Student’s *t* test; *p* values compared to MDCK: *** *p* = 0.0002 for Esh and *** *p* = 0.0005 for Esh+EEY; *p* values compared to ctrl sh: ** *p* = 0.0016 for Esh; * *p* = 0.0433 for Esh+EY, *** *p* = 0.0005 for Esh+EIY and *** *p* = 0.0001 for Esh+EEY. Esh 2 has significantly less Claudin-7 than MDCK and ctrl sh control lines; Esh+EY has Claudin-7 levels comparable to control lines; Esh+EIY and Esh+EEY have significantly more Claudin-7 than ctrl sh. **(E)** Claudin-7 immunoprecipitates (IP) of indicated MDCK cell lines were immunoblotted for EpCAM (top blot), YFP (middle blot) or Claudin-7 (bottom blot). ME in ctrl sh cells co-immunoprecipitates with Claudin-7 (top EpCAM blot) but ME is depleted in all Esh lines. EY in Esh+EY cells and EEY in Esh+EEY cells co-immunoprecipitate with Claudin-7 (top and middle blot for EY, middle blot for EEY). EEY is not recognized by EpCAM antibody in the EpCAM blot (E: top blot; see C, D) but by antibody to YFP (middle blot). EIY is mutated in the Claudin-7 interaction domain and does not co-immunoprecipitate with Claudin-7 from Esh+EIY cells (top and middle blot). Immunoglobulin heavy chain (Ig) from the Claudin-7 antibody used for the immunoprecipitation is recognized by secondary antibodies in the immunoblots. Immunoprecipitations were done twice and were repeated with an independent set of Esh lines showing the same results.

**Fig 7:**
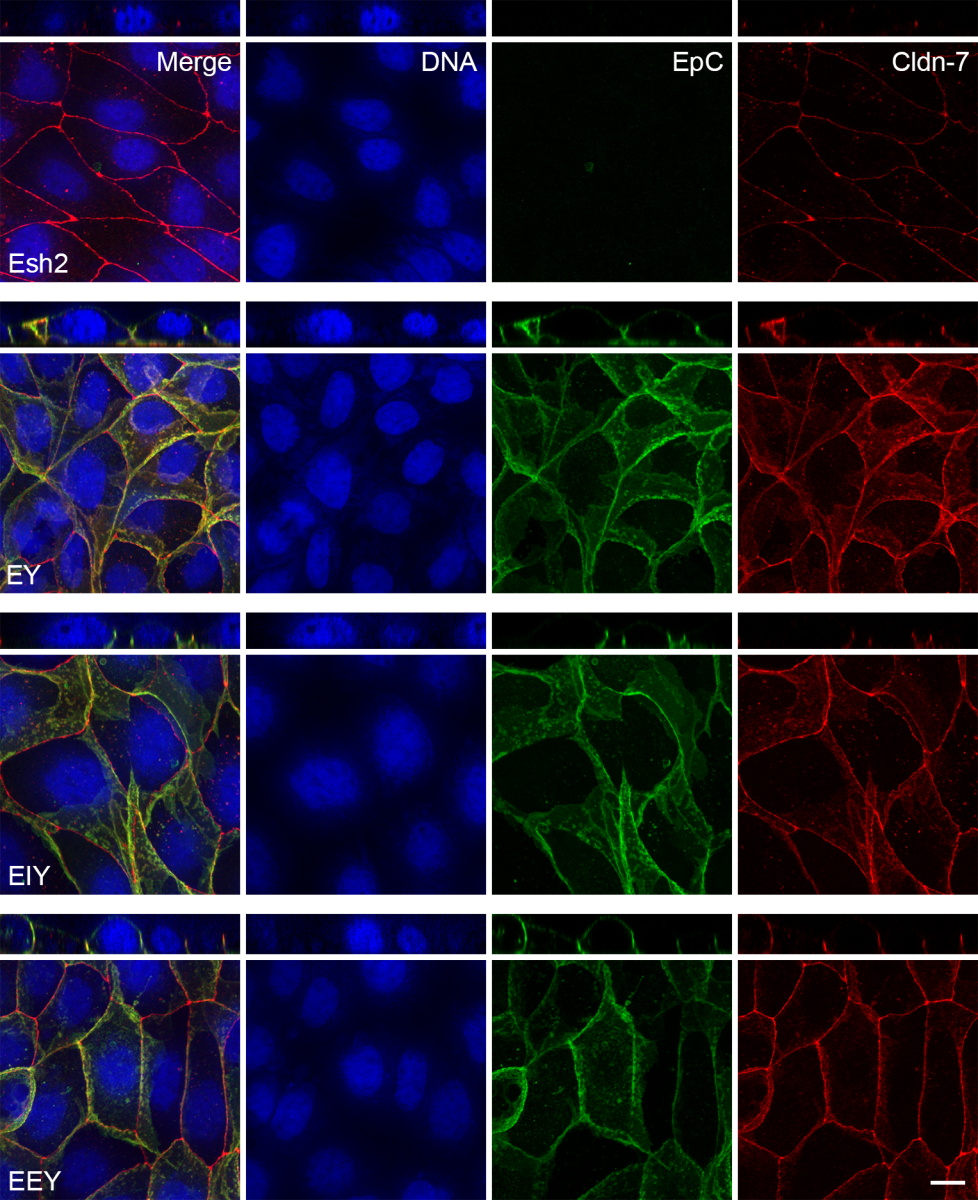
Expression of EpCAM-YFP constructs in Esh2 lines depleted of EpCAM rescues Claudin-7 localization to the basolateral membrane. Confocal images were acquired of cells 24 hours after high density plating on IBIDI chambers and 0 hours after removal of the chamber walls. The figure shows maximum intensity projections and orthogonal projections of confocal stacks of Esh2, EY, EIY and EEY cells stained for DNA, for YFP in the EpCAM-YFP fusion proteins and for Claudin-7. In the Esh2 line, Claudin-7 does not distribute along the basolateral membrane. In the EY, EIY and EEY expressing lines Claudin-7 localizes along the basolateral membrane. In the Esh2 line, greater contrast was applied post-processing to visualize the weaker Claudin-7 staining. In the EY, EIY and EEY the EpCAM-YFP fusion proteins localize along the basolateral membrane. Note, that Esh2 cells do not express any YFP-fusion protein and show no YFP-specific signal (see Fig 5C for anti-EpCAM staining of Esh2). Scale bar is 10μm.

Expression of EpCAM fused to Yellow fluorescent protein (YFP) at its C-terminus (Fig 6A and 6B: EY) in an MDCK cell line depleted of EpCAM (Fig 6C and 6D: Esh) rescues Claudin-7 protein levels in these cells (Fig 6C and 6D: Esh+EY) and also rescues Claudin-7 localization along the basolateral membrane (Fig 7: EY). EpCAM interaction with Claudin-7 depends on two amino acids—alanine and glycine—in an AxxxG motif in the transmembrane domain of EpCAM [6]. To inhibit interaction with Claudin-7, these amino acids were mutated to isoleucine in EpCAM-YFP “EIY” (Fig 6A). EIY protein localizes to the cell surface (Fig 6B) and rescues Claudin-7 protein levels in EpCAM-depleted MDCK cells (Fig 6C and 6D). However, EIY protein does not co-immunoprecipitate with Claudin-7 from cell lysates (Fig 6E), indicating that this mutant protein indeed cannot directly interact with Claudin-7 (Fig 6E) [6]. Confocal imaging shows that EIY protein localizes normally to the basolateral membrane, with a small level of expression on the apical surface. Furthermore, basolateral localization of Claudin-7 is rescued (Fig 7: EIY). These results indicate that direct protein-interaction of EpCAM and Claudin-7 is not required for regulation of Claudin-7 protein levels or for Claudin-7 localization to the basolateral membrane.

EpCAM interaction with Claudin-7 is inhibited by EpCAM dimerization and by cleavage of EpCAM by membrane-anchored serine protease matriptase [6,23]. Both dimerization and cleavage by matriptase depend on arginines R80 and R81 in the thyroglobulin type-1A (TY) domain of EpCAM [5,23]. The region around R80 and R81 forms a loop that protrudes from the otherwise compactly folded extracellular EpCAM domain [5], making it suitable for mutation without disrupting folding of the rest of the extracellular domain. To inhibit cis-dimerization and cleavage by matriptase, R80 and R81 were changed to E80 and E81 in EpCAM-YFP (Fig 6A: EEY) and EEY protein was expressed in EpCAM-depleted MDCK cells (Fig 6C and 6D: Esh+EEY). EEY localizes to the cell surface (Fig 6B), is very efficient in rescuing Claudin-7 protein levels in EpCAM-depleted MDCK cells (Fig 6C and 6D: Esh+EEY), and directly interacts with Claudin-7 (Fig 6E). Confocal imaging shows that EEY protein localizes to the basolateral membrane, with a small amount of apical expression, and that Claudin-7 localization is also basolateral (Fig 7: EEY). These results indicate that EpCAM dimerization is not required for regulation of Claudin-7 protein levels or for Claudin-7 localization to the basolateral membrane.

MDCK endogenous EpCAM appears as a single protein band in EpCAM immunoblots (Figs 1A, 2A and 6C), however, EpCAM-YFP proteins EY and EIY appear as double bands, indicating that these proteins may be processed by a cellular protease (Fig 6C: EpCAM and YFP blots). Double bands of EpCAM have been observed before and can be caused by activity of the cellular protease matriptase [23]. The origin of the double bands in the MDCK cell lines expressing EpCAM-YFP proteins EY, EIY and EEY is unclear. Both EEY protein bands appear to be slightly larger in immunoblots than the larger of the two EY and EIY proteins which may be caused by introduction of two negatively charged amino acids.

### The Claudin-7 interaction domain of EpCAM is required for regulation of ERK and epithelial migration

**Fig 8:**
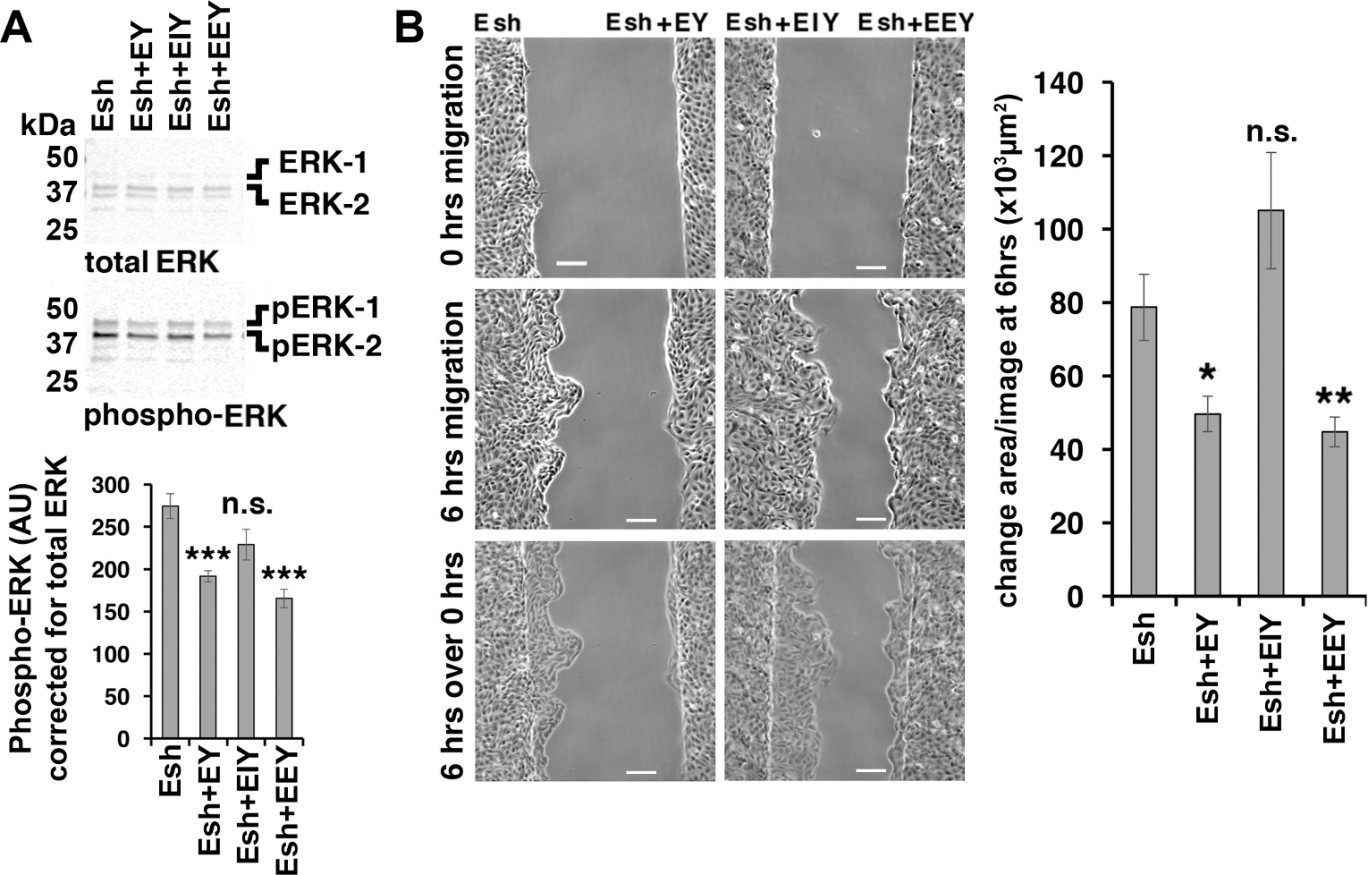
Rescue of epithelial sheet migration by expression of EpCAM-YFP or point mutants of EpCAM-YFP in MDCK cells depleted of EpCAM. **(A)** Phospho-ERK levels in EpCAM-depleted Esh2 cells (Esh) and Esh2-derived cell lines were analyzed as in Fig 5A and 5B. Error bars: S.E.M. of six independent samples for each cell line; *p* values derived from unpaired Student’s *t* test: *** *p* = 0.0004 for Esh+EY to Esh and *** *p* = 0.0001 for Esh+EEY to Esh. Phospho-ERK levels in Esh+EIY were not significantly reduced from the levels in the parental Esh line. **(B)** Phase images of indicated cell lines at 0 hrs (top row), and 6 hrs migration (middle row). The bottom row shows an overlay of “6 hrs” images onto “0 hrs” images. Scale bars: 100 μm. The graph in (B) shows quantification of average change of area/image after 6 hrs migration. Error bars: S.E.M. for five (Esh+EIY) to six experiments (Esh+EY, Esh+EEY) as shown in the phase images. Esh+EY and Esh+EEY migrate significantly slower than parental Esh cell sheets. The migration of Esh+EIY is not significantly different from Esh cell sheets. Stars correspond to *p* values derived from unpaired Student’s *t* test: * *p =* 0.0175 for Esh+EY and **p = 0.0063 Esh+EEY compared to Esh.

Expression of EpCAM-YFP “EY” or “EEY” rescues the increased ERK activation and increased migration in EpCAM-depleted “Esh” cells (Fig 8: Esh+EY and Esh+EEY compared to Esh for ERK activation in 8A and for migration in 8B). These results show that fusion of YFP to the intracellular C-terminus of EpCAM does not interfere with EpCAM’s regulation of ERK and epithelial sheet migration. These results also indicate that EpCAM-dimerization is not required for these functions because EEY is mutated in the extracellular cis-dimerization domain [5]. Since EpCAM dimerization is likely required for homophilic trans-interaction of EpCAM on the cell surface [5], EpCAM-mediated cell-cell adhesion may not be important for inhibition of ERK and epithelial migration. Please also note that work published during the review of our manuscript provides evidence that EpCAM is not mediating homophilic trans-interaction and is likely not a homophilic cell adhesion protein [38].

EpCAM mutant protein EIY does not rescue increased ERK activation or increased migration in EpCAM-depleted cells (Fig 8: Esh+EIY compared to Esh for ERK activation in 8A and for migration in 8B). The interaction domain for Claudin-7 in the transmembrane domain of EpCAM is defective in EIY and EIY does not bind to Claudin-7 (Fig 6E). Since EIY rescues Claudin-7 levels and localization in EpCAM-depleted cells (Fig 6C and 6D), these results indicate that rescue of Claudin-7 protein expression is insufficient for these functions but that EpCAM’s Claudin-7 interaction domain is required.

## Discussion

Our results show that loss of EpCAM raises the level of phospho-ERK, increases phospho-myosin in cortical F-actin, increases the number of phospho-myosin-rich leader cells, and speeds epithelial sheet migration. In contrast, elevating EpCAM levels in epithelial cells inhibits ERK and myosin activation, decreases the number of phospho-myosin-rich leader cells, and slows epithelial sheet migration. Importantly, the Claudin-7 interaction domain in the transmembrane region of EpCAM is required for regulation of ERK activity and epithelial sheet migration. In contrast, mutation of the extracellular domain of EpCAM that is required for cis-dimerization does not affect EpCAM’s regulation of ERK or sheet migration. Interestingly, disruption of EpCAM and Claudin-7 binding does not affect the levels or localization of Claudin-7, although loss of EpCAM depletes Claudin-7.

**Fig 9:**
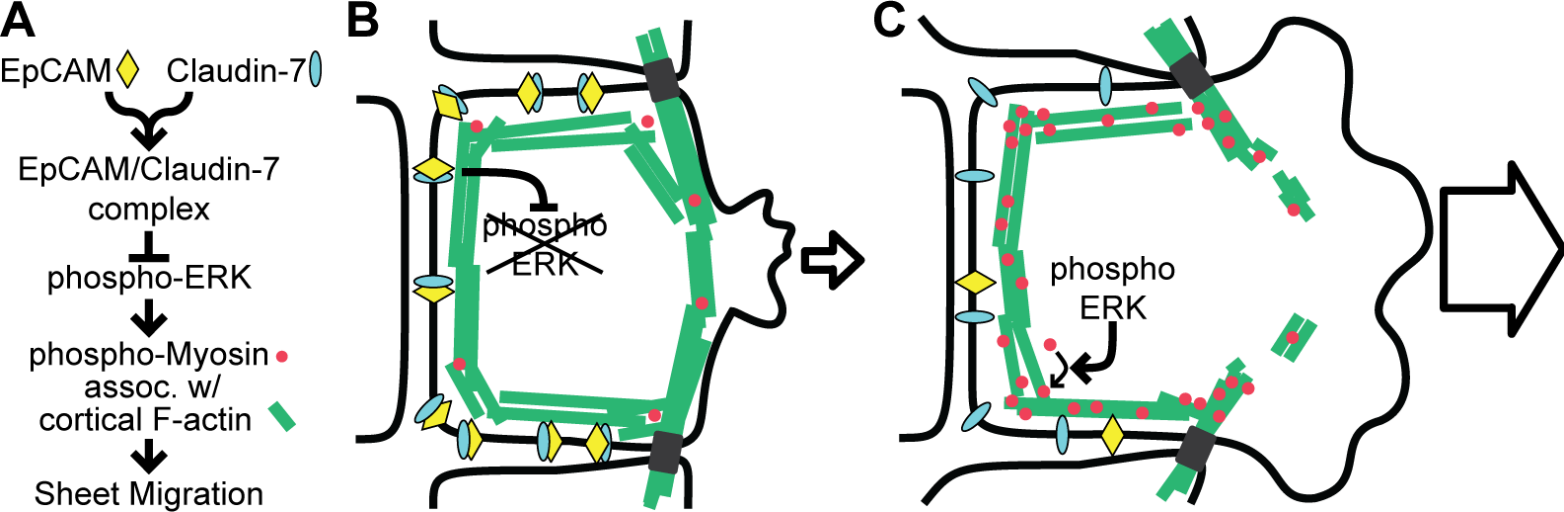
Interaction of EpCAM with Claudin-7 inhibits myosin activity in cortical F-actin and slows epithelial migration. (A) Proposed model of a pathway in which EpCAM/Claudin-7 complexes at cell-cell junctions inhibit ERK activity and myosin contractility in cortical F-actin. (B) Epithelial sheets with high EpCAM levels have low phospho-ERK levels and low phospho-myosin in cortical F-actin making it more difficult for cells at the edge to disrupt cortical F-actin bundles and extend lamellipodia. (C) Epithelial sheets with low EpCAM levels have high phospho-ERK levels and high phospho-myosin in cortical F-actin. Increased actomyosin contractility makes it easier for cells at the edge to disrupt cortical F-actin bundles and extend lamellipodia.

We propose a model where EpCAM/Claudin-7 complexes at cell-cell contacts provide a negative regulatory mechanism inhibiting actomyosin contractility, thereby inhibiting cell spreading and migration in response to migratory signals (Fig 9A). Direct EpCAM/Claudin-7 complexes at cell-cell junctions inhibit ERK phosphorylation and in turn actomyosin contractility in cortical F-actin and, finally, inhibit cell spreading and migration in response to migratory signals (Fig 9B). Conversely, in epithelial sheets with reduced levels of EpCAM, increased actomyosin contractility in cortical F-actin may help to disrupt cortical F-actin bundles and ease the protrusion of membrane into the gap resulting in extensive lamellipodia and an increased number of phospho-myosin-rich leader cells (Fig 9C).

Throughout this study, we have used an immortalized epithelial cell line, MDCK type II cells which forms polarized cell monolayers with well-established epithelial junctions in vitro [39]. In the following we will compare our results on EpCAM function in MDCK epithelial cells with results obtained in embryos *in vivo* or with results obtained with different cancer cell lines.

### Regulation of Claudin-7 by EpCAM

Loss of EpCAM in mice has been reported to cause loss of Claudin-7 protein from intestinal epithelium but not of Claudin-7 mRNA. This indicates that EpCAM regulates Claudin-7 post transcription [8]. Furthermore, EpCAM has been reported to protect Claudin-7 from endocytosis and degradation in lysosomes [6,22–24]. These results indicate that EpCAM regulates Claudin-7 levels by stabilizing it at the cell surface and that direct interaction between these proteins is required for Claudin-7 stabilization [6,8,22–24].

Accordingly, our results show that depletion of EpCAM causes a strong decrease of Claudin-7 protein levels in MDCK cells and loss of Claudin-7 can be rescued by expressing EpCAM-YFP. Unexpectedly, we also found that an EpCAM-YFP protein mutated in the Claudin-7 interaction domain rescued Claudin-7 levels and localization in EpCAM-depleted MDCK cells. This indicates that direct interaction of EpCAM with Claudin-7 through this domain is not required to regulate Claudin-7 protein levels and localization, and that there may be other mechanisms by which EpCAM regulates Claudin-7 protein expression in epithelial cells.

### The EpCAM/Claudin-7 complex as a regulator of ERK

Our results indicate that direct interaction of EpCAM with Claudin-7 is required to inhibit ERK activation and epithelial migration in MDCK cells. These results are supported by findings that EpCAM negatively regulates ERK activation during *Xenopus laevis* embryogenesis [13]. EpCAM also negatively regulates ERK activation and epithelial migration in a variety of mammalian epithelial cell lines [27].

Independent of EpCAM, it has been shown previously that Claudin-7 negatively regulates ERK activation and migration when expressed in a human lung carcinoma cell line [40]. In contrast to ours and these other previously published results, co-expression of EpCAM and Claudin-7 in human embryonic kidney (HEK) cells and in rat pancreatic adenocarcinoma (AS) cells increases ERK activity and cell migration [6]. Further experiments are needed to evaluate these differences of EpCAM function in embryos and in cancerous versus non-cancerous mammalian cell lines. It has been shown that EpCAM regulates cell differentiation during embryonic development and this function depends on cell-type and/or tissue specific activity of signaling pathways, such as Wnt signaling [12,14]. These pathways may not be active in differentiated epithelial cells but are reactivated in many cancers [41]. Therefore, it is important to analyze EpCAM function in the context of its cellular environment.

### ERK as a regulator of actomyosin contractility and cell migration

ERK has been shown previously to activate myosin light chain kinase and to increase phospho-myosin levels and actomyosin contractility [13,33]. Accordingly, we observed reduced phospho-myosin levels along cortical F-actin in MDCK cells with increased EpCAM and decreased ERK activation. Conversely, loss of EpCAM has been associated with increased phospho-myosin levels and increased actomyosin contractility [11,13] and consistent with these previous published results we found that phospho-myosin was enriched in cortical F-actin and especially in multicellular junctions between EpCAM-depleted MDCK cells. Increased phospho-myosin in cortical F-actin leads to destabilization of cell-cell adhesion and reduced levels of C-cadherin in the ectoderm of *X*. *laevis* embryos [13]. In low density cultures of EpCAM-depleted MDCK cells gaps were present between cells at some of these phospho-myosin-rich multicellular junctions, which may have been caused by loss of cell-cell adhesion due to increased actomyosin contractility in these areas [42].

In summary, our results suggest a pathway in which EpCAM/Claudin-7 complexes at the cell surface negatively regulate ERK-mediated myosin phosphorylation and actomyosin contractility, thereby slowing epithelial sheet migration (Fig 9). These results give new insight into the mechanism by which abnormal EpCAM expression can cause disease. For example, EpCAM mutations can cause congenital tufting enteropathy (CTE), in which abnormal actomyosin contractility at tricellular junctions causes extension of the apical domain and pathological tuft formation of the intestinal epithelium [11]. Our results show that the interaction domain of EpCAM with Claudin-7 is important to maintain normal epithelial morphology by regulating actomyosin contractility at junctions. Ultimately, insight into EpCAM’s protein interactions can drive a deeper understanding of EpCAM in various contexts ranging from diseases such as CTE and cancer to developmental processes.

## Material and methods

### Cell lines

Growth conditions for Madin-Darby canine kidney (MDCK) type II/G cells have been described previously [43]. MDCK cells have been transfected with DNA constructs using lipofectamine 3000 reagent as described by the manufacturer (Gibco BRL, Gaithersburg, MD). One day after transfection cells were selected three days with 1mg/ml G418 or with 10 μg/ml puromycin. Subsequently, cells were cultivated in 300μg/ml G418 or 3μg/ml puromycin, respectively. Two to three weeks after transfection, G418-resistant cells expressing human EpCAM were stained with anti-CD326 (EpCAM)-PE (MACS Miltenyi Biotec Inc., Auburn, CA) as described by the manufacturer and single cells were sorted into 96 wells by fluorescence-activated cell sorting (Stanford Shared FACS Facility, Stanford University, Stanford, CA).

Puromycin-resistant control and EpCAM shRNA expressing cells were sorted for co-expression of Cherry fluorescent protein from expression vector psi-mU6 (GeneCopoeia, MD; CS-SH134T-mU6-01). Stable MDCK cell lines either overexpressing EpCAM or depleted of EpCAM were established by screening clonal lines for EpCAM levels by immunoblotting with anti-EpCAM antibody UMAB131 (OriGene Technologies, Inc., Rockville, MD). EpCAM-depleted MDCK line Esh2 was transfected with expression vectors for EY (EpCAM-YFP), EIY (EpCAM-YFP defective for Claudin-7 binding), or EEY (EpCAM-YFP mutated in matriptase cleavage and dimerization domain). Expressing cells were initially selected with 500 μg/ml G418 and 3 μg/ml Puromycin and subsequently cultivated in 200 μg/ml G418 and 2 μg/ml Puromycin and sorted by FACS for expression of Cherry and Yellow fluorescent proteins as described above (Stanford Shared FACS Facility, Stanford University, Stanford, CA). All lines were cultured at least 24 hours in the absence of antibiotics before experiments were performed.

### DNA constructs

For the generation of MDCK cell lines MhE16 and MhE33 overexpressing EpCAM, EpCAM expression vector SC322331 was obtained from OriGene Technologies, MD. The human EpCAM cDNA insert (NCBI accession no. NM_002354) in expression vector pCMV6-AC was confirmed by sequencing (Sequetech Corporation, CA).

To design shRNA vectors specific for the depletion of MDCK EpCAM mRNA, total RNA was extracted from MDCK cells and fragments of EpCAM mRNA were amplified by RT-PCR. Forward primers TTGGTGCACAAAATTCTGTCATTTGCTCAAAACTGGC; GCGAATGTACTTCAATTGGTGCACAAAATTCTGTCATTTGCTC; CTGGCAACCAAATGTTTGGTTATGAAGGCAGAAATGACCGGC; GTGCTGGTGTGTGAACACTGCTGGGGTCCGAAGAAC and reverse primer TGCATTGAGTTCCCTATGCATCTCACCCATCTC were designed using the sequence of predicted *Canis lupus familiaris* EpCAM mRNA (NCBI accession no. XM_005626238.1). Independent RT-PCR products derived from MDCK RNA were sequenced. These MDCK-derived sequences were identical with the predicted *Canis lupus familiaris* EpCAM mRNA and used to design the following shRNA vectors: EpCAM shRNA 1: ACCACAAACTGCTCTTTGAAC; EpCAM shRNA 2: GACGAATCCTTGTTCCATTCC in psi-mU6 (GeneCopoeia, MD; CS-SH134T-mU6-01). EpCAM shRNA vectors were used to generate MDCK lines Esh1 and Esh2, respectively by FACS sorting for co-expression of Cherry as described above. The GeneCopoeia shRNA scrambled control in psi-mU6 was used to establish the MDCK line ctrl sh (GeneCopoeia, MD; CSHCTR001-mU6).

For the rescue of EpCAM function in EpCAM-depleted MDCK line Esh2 the following vectors were constructed by Epoch Life Science Inc., TX: the cDNA sequence for enhanced Yellow Fluorescent Protein (modified from pEYFP, Clontech, CA) was fused to the 3’ end of the cDNA sequence for human EpCAM in vector pCMV6-AC (OriGene Technologies, MD, product SC322331) to obtain the EpCAM-YFP expression vector for EY. To generate the expression vector for EEY, two single nucleotide exchanges were introduced in the EY vector into the arginine codons for R80 and R81 (human EpCAM cDNA, NCBI accession no. NM_002354) to change them into codons for glutamates E80 and E81, respectively. To generate the expression vector for EIY, two single nucleotide exchanges were introduced into the codons for alanine A278 and glycine G282 (human EpCAM cDNA, NCBI accession no. NM_002354) to change them into codons for isoleucines I278 and I282, respectively. EpCAM cDNA inserts in these expression vectors were confirmed by sequencing (Sequetech Corporation, CA). Canine-specific EpCAM shRNA 2 has four mismatches to the corresponding sequence in human EpCAM cDNA and does not inhibit expression of these human EpCAM-YFP proteins in MDCK line Esh2.

### Time lapse microscopy of epithelial sheet migration

For epithelial sheet migration assays in Figs 1, 2 and 8, cell lines were plated at confluent density (5x10^4^ cells per well). MDCK and EpCAM-overexpressing MhE16 cells, or ctrl sh cells and EpCAM-depleted Esh2 cells were plated into one of each well on the same IBIDI dish with 2 or 4-well culture inserts (Thermo Fisher Scientific, MA). Wells were removed after 24 hours, medium was exchanged with phenol-red-free DMEM, 10% FBS, 25 mM HEPES pH 7 and dishes were immediately set up for multi-site imaging of 12 to 24 sites along the edge of epithelial sheets. Removal of chamber walls introduced a gap of 500 μm width between epithelial sheets and induced migration of epithelial sheets into these gaps. The images in Figs 1C, 2C and 8B show the full width of the gap and the edges of epithelial sheets at both sites of the gap. For each experiment images were taken along the full length of all 500 μm gaps between epithelial sheets. In Figs 1C, 2C and 8B the “0 hour” time point of migration corresponds to the first image of the time lapse which was taken 30 min after removal of chamber walls. Migration into the gap was imaged overnight at 10 minute intervals. All migration assays were performed with a customized Zeiss Observer.Z1 inverted microscope controlled by Marianas Slidebook 6 software (Intelligent Imaging Innovations, 3I, CO) using a Zeiss Plan Apochromat Phase-1 10x/0.25 objective in a temperature- and CO_2_-controlled incubator and images were collected with a Hamamatsu Orca C9100 EM-CCD.

Quantification of sheet migration was performed using ImageJ macros. First, the images of the sheets were converted into binary images using the following sequence of ImageJ commands: Find Edges, Subtract Background, Enhance Local Contrast (CLAHE), Threshold (Default), Fill Holes, Dilate, Close, Dilate. The binary images were used to measure the area covered by each sheet in each frame. The area migrated at each time-point was calculated by subtracting the area covered in the first frame from the area covered in the current frame. For each experiment, the monolayer boundary was captured using 11–12 images, and the average of the 12 sites for each line was taken as a single data point for analysis since the cells for a given experiment are highly correlated in their migration with their neighbors. Nine experiments were carried out for the MDCK line, four for the ctrl sh, four for the Esh1, three for the Esh2, four for the MhE16, five for the MhE33, six for the Esh+EY, five for the Esh+EIY, and six for the Esh+EEY. Significance was calculated using an unpaired Student’s *t*-test.

### Cell surface area measurement

For phase images in Figs 1B and 2B, cells were grown at low density without antibiotics for two days on collagen-coated coverslips. These cells were then incubated with 1:1000 Hoechst 33342 (AnaSpec Inc., CA) for 30 minutes. The media was exchanged for imaging media and imaged on a Leica DMI6000B inverted microscope with a Leica PH1 Semi-Apochromat 10x/0.3 objective, and a QImaging Retiga 2000R CCD camera. Images were taken of each line using both phase-contrast, and blue fluorescence (Leica A4 filter cube), with multiple colonies in each image. To quantify the area per cell, the phase-contrast and blue fluorescence images were converted to binary images using custom automated ImageJ macros. The phase contrast images were binarized using the following ImageJ commands: Subtract Background, Enhance Local Contrast (CLAHE), Kuwahara Filter, Threshold (Huang), Fill Holes, Dilate, Close, Fill Holes, Erode, Gaussian Blur. Only colonies that were not touching the edge of the field-of-view were analyzed. The thresholded colonies were then used to crop the nuclei images, which were then binarized using the following ImageJ commands: Subtract Background, Enhance Local Contrast (CLAHE), Gaussian Blur, Threshold (Default), Watershed. Cell area per nuclei was calculated by dividing the area of the colony mask by the number of nuclei within the colony mask. The data was then manually checked for errors in detection of colonies or nuclei. 90 MDCK, 88 ctrl sh, 104 Esh1, 58 Esh2, 268 MhE16 and 284 MhE33 small colonies of 5 to 200 cells were analyzed. Significance was calculated using a two-tailed Mann Whitney U-test.

### Immunoprecipitation

Immunoprecipitation of Claudin-7 protein complexes were done as described previously for other protein complexes [44] with the following variations: MDCK cells were plated at confluent density on Falcon cell culture inserts (Thermo Fisher Scientific, MA), after one day washed in 10 mM Tris-HCl pH7, 140 mM NaCl and extracted in cold lysis buffer: 10 mM Tris-HCl, pH 7, 140 mM NaCl, 300 mM sucrose, 0.5% Triton-X-100, cOmplete EDTA-free protease inhibitor cocktail and PhosSTOP phosphatase inhibitor cocktail (Roche Diagnostics GmbH, Mannheim, Germany). Cell lysates were precleared with Protein A-conjugated Sepharose-4B (Sigma) and incubated 16 hours at 4°C with rabbit anti-Claudin-7 antibody (Thermo Fisher Scientific, MA). Protein A-conjugated Sepharose-4B was added to lysates for one hour, protein A-bound antibody/protein complexes were precipitated by centrifugation. Immunoprecipitates were washed three times with 30x volume lysis buffer at 4°C, boiled 5 min. at 100°C in Laemmli SDS sample buffer and immunoblotted as described below.

### Immunoblotting and quantitation of protein levels

SDS PAGE and western blotting was done as described previously [43] with the following variation: protein samples were separated in 4–20% Mini-Protean precast protein gels (Bio-Rad, CA). The following antibodies and dilutions were used dilution 1:500: mouse monoclonal anti-EpCAM antibody UMAB131 (OriGene Technology, MD); mouse anti-ERK and rabbit anti-phospho-ERK (Cell Signaling Technology, MA); rabbit anti-Claudin-7 34–9100, anti-Claudin-1 51–9000, and anti-Claudin-3 34–1700 (Thermo Fisher Scientific, MA); mouse anti-GFP (Roche Diagnostics GmbH, Mannheim, Germany); rabbit E-cadherin 24E10 (Cell Signaling Technology, MA) and mouse anti-GAPDH at 1:1000 (Abcam, MA). Secondary goat antibodies against rabbit or mouse IgG with minimal cross-reactivity were coupled to either IRDye 680LT (LI-COR Biotechnology, Lincoln, NE) or Alexa Fluor 680 (Thermo Fisher Scientific, MA) and used at a dilution of 1:10,000. Immunoblots were scanned at 680 nm and 800 nm using an Odyssey infrared imaging system (Li-COR Biotechnology, Lincoln, NE) and quantified with Odyssey software (LI-COR Biotechnology, Lincoln, NE). To quantify ERK phosphorylation, the intensity of the pERK protein bands was normalized to the total ERK intensity detected independently in the same sample in the same lane (Figs 3, 8 and S1). To obtain normalization factors for the total ERK signals, one of the lanes with MDCK sample was designated as having 1x ERK level and total ERK levels in the other samples of the same immunoblot were calculated in comparison to this MDCK sample. Phospho-ERK levels were calculated by dividing the pERK signal in each lane by the ERK normalization factor obtained for the same lane. Therefore, the pERK values shown in the graphs in Figs 3, 8 and S1 correspond to the pERK/ERK ratios of each sample. This analysis corrects for differences in total ERK levels between samples and allows to compare pERK/ERK ratios between cell lines, but it will not allow to measure differences in total ERK levels between cell lines. To quantify the protein levels of total ERK (S1A” Fig), EpCAM (Figs 1, 2 and 6), Claudin-7 (Figs 5 and 6), Claudins -1 and 3 (S5 Fig), and E-cadherin (S6 Fig), the intensity of the bands was normalized to GAPDH protein levels in the same sample. Significance was calculated using unpaired Student’s *t* test.

### Cell fixation, antibodies and fluorescence imaging

For widefield fluorescence images in Fig 4A and 4B, cells were grown without antibiotics at low density for two days on collagen-coated coverslips. For fluorescence images in Fig 4D, cells were plated as for migration assays in Figs 1 and 2 at confluent density (5x10^4^ cells per well). Control MDCK and ctrl sh cells, EpCAM-overexpressing MhE16 cells, and EpCAM-depleted Esh2 cells were plated into one of each well on the same IBIDI dish with 4-well culture insert (Thermo Fisher Scientific, MA), wells were removed after 24 hours and cells were fixed after 6 hours of migration as described above. The cells were fixed in 4% Formaldehyde, 0.4% Triton-X-100 in Dulbecco’s phosphate buffered saline and labelled with the following reagents and antibodies as described previously [44,45]: rabbit polyclonal phospho-myosin light chain 2 (Ser19) antibody 1:100 (Cell Signaling Technologies, MA), Alexa Fluor 488 Phalloidin 1:100 (Cell Signaling Technologies, MA), secondary Alexa Fluor 647 goat anti-Rabbit SFX Kit, highly cross-adsorbed 1:100 (Thermo Fisher Scientific, MA) and mounting medium Vectashield with DAPI (Vector Laboratories, CA).

Widefield images were acquired using a Zeiss Axiovert 200M microscope with a Zeiss Plan Neofluar 40x/1.3 oil Phase-3 objective or a Zeiss Plan-Apochromat 63x/1.4 oil DIC objective (Carl Zeiss Meditec, Dublin, CA) with the following filter set: DAPI (Chroma 31000v2), FITC (Omega XF22), Rhodamine (Omega XF37), Cy5 (Chroma 41008). Images were recorded using Micro-Manager 1.4 software (University of California, San Francisco, CA) with identical exposure times for each fluorescence staining and fluorescence intensities were analyzed using ImageJ (National Institutes of Health, MD).For confocal fluorescence images in Figs 5C and 7, and S3 and S4 Figs, cells were plated at confluent density (5x10^4^ cells per well) on an IBIDI dish with 4-well culture inserts (Thermo Fisher Scientific, MA) and grown for 24 hours without antibiotics. The inserts were removed, and the cells were immediately fixed with ice-cold methanol, taking care not to wash off the cells. The MDCK and MhE16 cells were labeled as previously described [44,45] with mouse monoclonal anti-EpCAM UMAB131 1:100 (Origene, MD), rabbit polyclonal anti-Claudin-7 1:100 (Invitrogen, CA), mouse anti-ZO1 ZO1-1A12 1:100 (Thermo Fisher Scientific, MA), secondary goat anti-Mouse AffiniPure FITC 1:100 (Jackson ImmunoResearch Laboratories, PA), and Alexa Fluor 647 goat anti-Rabbit SFX Kit, highly cross-adsorbed 1:100 (Thermo Fisher Scientific, MA). The Esh2, EY, EIY and EEY cells were labeled with chicken polyclonal anti-GFP 1:100 (GeneTex, CA), rabbit polyclonal anti-Claudin-7 1:100 (Invitrogen, CA), mouse anti-ZO1 ZO1-1A12 1:100 (Thermo Fisher Scientific, MA), secondary Alexa Fluor 488 goat anti-Chicken 1:100 (Thermo Fisher Scientific, MA), secondary goat anti-Mouse Rhodamine Red-X 1:100 (Jackson ImmunoResearch Laboratories), and secondary Alexa Fluor 647 goat anti-Rabbit SFX Kit, highly cross-adsorbed 1:100 (Thermo Fisher Scientific, MA). All confocal samples were additionally stained with Hoechst 33342 1:10,000 (AnaSpec Inc., CA), and mounted with SlowFade Diamond Antifade Mountant (Invitrogen, CA). Confocal images were taken on a Zeiss LSM780 using a Plan Apo 1.4NA 63x oil immersion objective. For confocal images, the Esh2, EY, EIY, and EEY cells were not stained or imaged under the same conditions as the MDCK and MhE16 due to the expression of fluorescent proteins (EpCAM-YFP and mCherry) in the Esh2 and derivative lines.

### Phospho-myosin/F-actin and leader cell quantitation

For Fig 4C, the ratios of phospho-myosin to F-actin shown in Fig 4A and 4B were measured by dividing the mean phospho-myosin intensity by the mean F-actin intensity within a region of interest defined by the cortical F-actin. The regions of interest were generated using the F-actin channel and the following sequence of ImageJ commands: Background Subtraction, Threshold (Moments), and Despeckle. The ROIs were then used to mask the phospho-myosin and F-actin channels respectively. The mean value of the masked images was then calculated using built-in ImageJ commands, and exported as a csv for further processing. The phospho-myosin to F-actin ratio was calculated as the ratio of the mean value of the two channels. A random region corresponding to an area with no cells was used to subtract the background fluorescence in each channel. At least 30 independent images per cell line were analyzed. Significance was calculated with a two-tailed Mann Whitney U-test.

For the graph in Fig 4E, the total number of leader cells along the edge of epithelial sheets originating from each well at 6 hours of migration was manually quantified. To determine if a cell was a leader cell we looked for the following conditions: presence of lamellipodial protrusions, a break in cortical actin cable, and a greater displacement towards the open wound area relative to neighboring cells. The entire perimeter of the monolayer was considered for the counts. Four independent monolayers for each cell line were quantified. Significance was calculated using an unpaired Student’s *t*-test.

To measure the percent edge of the sheet with lamellipodium in Fig 4F, for each edge roughly 20 images of the monolayer boundary were taken in the phalloidin channel using a 20x objective and a custom Matlab script. The tiled images were stitched together using an ImageJ Grid/Collection Stitching plugin [46]. To measure the total length of the leading edge the images were thresholded, the total perimeter of the edge was measured using the ImageJ wand tool, and the line segments corresponding to the image boundaries were manually traced and subtracted from the total perimeter. To measure the total length with lamellipodia, the regions of the edge with lamellipodia were manually traced. Finally, the % edge lamellipodia was calculated as the total length of lamellipodia divided by the total length of the leading edge. Four independent monolayer boundaries from different dishes for each cell line were quantified. Significance was calculated using an unpaired Student’s *t*-test.

## Supporting information

S1 Fig EpCAM inhibits ERK activation in response to Hepatocyte growth factor (HGF). **(A)** Wildtype MDCK, and MDCK lines overexpressing human EpCAM (MhE16, MhE33) were SDS extracted one day after plating at subconfluent cell density; levels of ERK and phospho-ERK were analyzed in the same immunoblot. **(A’)** The graph shows quantification of combined 44 and 42 kDa phospho-ERK protein levels normalized to combined 44 and 42 kDa ERK protein levels in the same sample. Error bars: S.E.M. of three independent samples for each cell line; *p* values derived from unpaired Student’s *t* test: *** *p* = 0.0004 for MhE16 to MDCK and * *p* = 0.0128 for MhE33 to MDCK. **(A”)** The graph shows quantification of combined 44 and 42 kDa total ERK protein levels normalized to GAPDH protein levels in the same sample. Error bars: S.E.M. of three independent samples for each cell line; total ERK levels are not significantly different in these three cell lines. **(B-D)** MDCK cells and MDCK cell lines MhE16, MhE33 overexpressing human EpCAM were plated at low density for one day, serum-starved for 2 hours and extracted **(B)**, or serum-starved for 2 hours and treated with 5 ng/ml HGF for 5 minutes **(C)** or 60 minutes **(D)**. Cells were SDS extracted and levels of ERK and phospho-ERK were analyzed in the same immunoblot. **(B’-D’)** The graphs show quantification of combined 44 and 42 kDa phospho-ERK protein levels normalized to combined 44 and 42 kDa ERK protein levels in the same sample. Arbitrary units for protein intensities in Y-axis (AU) x10^3^; error bars: S.E.M. of three independent samples for each cell line; *, **p values compared to MDCK cells derived from unpaired Student’s *t* test. In (B’) MhE16 **p = 0.0049, MhE33 *p = 0.0179; in (C’) MhE16 *p = 0.0161, MhE33 *p = 0.0288; in (D’) MhE16 **p = 0.0038, MhE33 **p = 0.0057. **(E)** Phospho-ERK levels from graphs of serum-starved (0) cells in (B’), or cells treated 5 minutes (C’) or 60 minutes (D’) with HGF are combined into one graph in (E) to compare HGF-induced phospho-ERK activation over time in these cell lines. Arbitrary units for protein intensities in Y-axis (AU) x10^3^; error bars: S.E.M. of three independent samples for each time point for each cell line. **(F)** Phospho-ERK protein intensities measured in (D) are represented as fold activation compared to phospho-ERK intensities in serum-starved cells for each line (0 minutes HGF). Phospho-ERK protein levels are lower in serum-starved MhE cells (B') and remain lower compared to control MDCK cells at 5 minutes (C', E) or 60 minutes (D', E) of treatment with HGF. However, fold activation of ERK normalized to baseline levels in serum-starved cells is similar (F).
(TIF)

S2 Fig Phospho-myosin and cortical F-actin levels in smaller colonies. **(A)** Examples of smaller colonies of cells cultured and images as described in Fig 4A. Phospho-myosin-rich areas of cortical F-actin at the edge of colonies are marked with arrows and phospho-myosin-rich multicellular junctions inside colonies are marked with arrowheads. Bars = 50μm.
(TIF)

S3 Fig Confocal images of ZO-1 and Claudin-7 localization in MDCK and MhE lines. Confocal images of cells prepared as in Fig 5C, stained for nuclei (blue) tight-junction marker ZO-1 (green) and Claudin-7 (red). In both MDCK and MhE16 lines, Claudin-7 localizes along the entired basolateral membrane, whereas ZO-1 is restricted to the apical side of the lateral membrane corresponding to the tight junctions. Scale bar is 10μm.
(TIF)

S4 Fig Confocal images of ZO-1 and Claudin-7 localization in Esh2, EY, EIY and EIY lines. Confocal images of cells prepared as in Fig 5C, stained for nuclei (blue) tight-junction marker ZO-1 (green) and Claudin-7 (red). In the Esh2 line, Claudin-7 colocalizes with the ZO-1 and is restricted to the apical side of the lateral membrane corresponding to the tight junctions. In the EY, EIY and EEY lines the Claudin-7 localization is rescued and once again distributes along the basolateral membrane, while ZO-1 remains restricted to the tight junctions. Scale bar is 10μm.
(TIF)

S5 Fig Claudin-1 and -3 protein levels in EpCAM-depleted or over-expressing MDCK cell lines. **(A)** Claudin-1 and **(B)** Claudin-3 protein levels were analyzed as described for Claudin-7 in Fig 6. Graphs show quantifications of indicated proteins normalized to GAPDH levels in the same sample. Arbitrary units for protein intensities in Y-axis (AU) x10^3^; error bars: S.E.M. of six samples for each cell line. Protein extracts are the same as in Fig 6. **(A)** Expression of Claudin-1 is only slightly reduced, **(B)** expression of Claudin-3 is more strongly reduced in EpCAM-depleted Esh2 cells (Esh) compared to MDCK and control shRNA line (ctrl sh). Expression of EY, EIY or EEY in Esh2 rescues Claudin-1 and -3 protein levels in these cell lines compared to levels in the Esh line. Higher EpCAM and Claudin-7 levels in the EIY and EEY lines compared to parental MDCK or ctrl sh control lines (see Fig 6).
(TIF)

S6 Fig E-Cadherin protein levels in EpCAM-depleted or over-expressing MDCK cell lines. The same protein extracts as shown in Fig 5A and 5B, respectively, were immunoblotted for E-Cadherin and E-cadherin levels were normalized for GAPDH. Arbitrary units for protein intensities in Y-axis (AU) x10^3^; error bars: S.E.M. of four samples for each cell line in **(A)** and three samples for each cell line in **(B);** *p* values derived from unpaired Student’s *t* test: ** *p* = 0.0055 for Esh2 to ctrl sh; * *p* = 0.025 for MhE16 to MDCK and * *p* = 0.016 for MhE33 to MDCK. EpCAM-depleted Esh2 MDCK cells have slightly more E-cadherin **(A)** whereas EpCAM-overexpressing MDCK cells have slightly less E-cadherin **(B).**
(TIF)

S1 Movie EpCAM-depleted MDCK cell line E sh 1 migrates faster than parental MDCK cells. 600X time-lapse with images taken at 10 minute intervals. Quantification of this time lapse is shown in Fig 1C.
(AVI)

S2 Movie EpCAM-depleted MDCK cell line E sh 2 migrates faster than MDCK cells expressing a control sh RNA (ctrl sh). 600x time-lapse with images taken at 10 minute intervals. Quantification of this time lapse is shown in Fig 1C.
(AVI)

S3 Movie EpCAM-overexpressing MDCK cell line MhE16 migrates faster than MDCK cells. 600x time-lapse with images taken at 10 minute intervals. Quantification of this time lapse is shown in Fig 2C.
(AVI)

S4 Movie EpCAM-overexpressing MDCK cell line MhE33 migrates faster than MDCK cells. 600x time-lapse with images taken at 10 minute intervals. Quantification of this time lapse is shown in Fig 2C.
(AVI)

S5 Movie Migration is slowed down by expression of EpCAM-YFP in EpCAM-depleted MDCK cell line Esh2 (Esh+EY). 600x time-lapse with images taken at 10 minute intervals. Quantification of this time lapse is shown in Fig 7B.
(AVI)

S6 Movie Migration in EpCAM-depleted MDCK cell line Esh2 is slowed down by expression of EpCAM-YFP with a mutation in the extracellular dimerization domain (Esh+EEY) but not by expression of EpCAM-YFP with a mutation in the transmembrane Claudin-7 interaction domain (Esh+EIY). 600x time-lapse with images taken at 10 minute intervals. Quantification of this time lapse is shown in Fig 7B.
(AVI)

## References

[1] Balzar M, Briaire-de Bruijn IH, Rees-Bakker HAM, Prins FA, Helfrich W, de Leij L, et al Epidermal Growth Factor-Like Repeats Mediate Lateral and Reciprocal Interactions of Ep-CAM Molecules in Homophilic Adhesions. Mol Cell Biol. 2001;21: 2570–2580. 10.1128/MCB.21.7.2570-2580.2001 11259604 PMC86888

[2] Litvinov S V, Balzar M, Winter MJ, Bakker HAM, Bruijn IHB, Prins F, et al Epithelial Cell Adhesion Molecule (Ep-CAM) Modulates Cell–Cell Interactions Mediated by Classic Cadherins. J Cell Biol. 1997;139: 1–12. 10.1083/jcb.139.5.1337 9382878 PMC2140211

[3] Litvinov S V., Velders MP, Bakker HAM, Fleuren GJ, Warnaar SO. Ep-CAM: A Human Epithelial Antigen is a Homophilic Cell-Cell Adhesion Molecule. J Cell Biol. 1994;125: 437–446. 10.1083/jcb.125.2.437 8163559 PMC2120036

[4] Trebak M, Begg GE, Chong JM, Kanazireva E V., Herlyn D, Speicher DW. Oligomeric State of the Colon Carcinoma-associated Glycoprotein GA733-2 (Ep-CAM/EGP40) and Its Role in GA733-mediated Homotypic Cell-Cell Adhesion. J Biol Chem. 2001;276: 2299–2309. 10.1074/jbc.M004770200 11058587

[5] Pavšič M, Gunčar G, Djinovi-Carugo K, Lenarčič B. Crystal structure and its bearing towards an understanding of key biological functions of EpCAM. Nat Commun. 2014;5 10.1038/ncomms5764 25163760

[6] Nubel T, Preobraschenski J, Tuncay H, Weiss T, Kuhn S, Ladwein M, et al Claudin-7 Regulates EpCAM-Mediated Functions in Tumor Progression. Mol Cancer Res. 2009;7: 285–299. 10.1158/1541-7786.MCR-08-0200 19276185

[7] Guerra E, Lattanzio R, la Sorda R, Dini F, Tiboni GM, Piantelli M, et al mTrop1/Epcam Knockout Mice Develop Congenital Tufting Enteropathy through Dysregulation of Intestinal E-cadherin/β-catenin. PLoS One. 2012;7 10.1371/journal.pone.0049302 23209569 PMC3509129

[8] Lei Z, Maeda T, Tamura A, Nakamura T, Yamazaki Y, Shiratori H, et al EpCAM contributes to formation of functional tight junction in the intestinal epithelium by recruiting claudin proteins. Dev Biol. Elsevier; 2012;371: 136–145. 10.1016/j.ydbio.2012.07.005 22819673

[9] Schnell U, Kuipers J, Mueller JL, Veenstra-Algra A, Sivagnanam M, Giepmans BNG. Absence of cell-surface EpCAM in congenital tufting enteropathy. Hum Mol Genet. 2013;22: 2566–2571. 10.1093/hmg/ddt105 23462293 PMC3674798

[10] Sivagnanam M, Mueller JL, Lee H, Chen Z, Nelson SF, Turner D, et al Identification of EpCAM as the Gene for Congenital Tufting Enteropathy. Gastroenterology. 2008;135: 429–437. 10.1053/j.gastro.2008.05.036 18572020 PMC2574708

[11] Salomon J, Gaston C, Magescas J, Duvauchelle B, Canioni D, Sengmanivong L, et al Contractile forces at tricellular contacts modulate epithelial organization and monolayer integrity. Nat Commun. 2017;8 10.1038/ncomms13998 28084299 PMC5241865

[12] Lu H, Ma J, Yang Y, Shi W, Luo L. EpCAM Is an Endoderm-Specific Wnt Derepressor that Licenses Hepatic Development. Dev Cell. Elsevier Inc.; 2013;24: 543–553. 10.1016/j.devcel.2013.01.021 23484855

[13] Maghzal N, Kayali HA, Rohani N, Kajava A V., Fagotto F. EpCAM Controls Actomyosin Contractility and Cell Adhesion by Direct Inhibition of PKC. Dev Cell. 2013;27: 263–277. 10.1016/j.devcel.2013.10.003 24183651

[14] Sarrach S, Huang Y, Niedermeyer S, Hachmeister M, Fischer L, Gille S, et al Spatiotemporal patterning of EpCAM is important for murine embryonic endo- and mesodermal differentiation. Sci Rep. Springer US; 2018;8: 1–18. 10.1038/s41598-017-17765-5 29379062 PMC5789065

[15] Allard WJ, Matera J, Miller MC, Repollet M, Connelly MC, Rao C, et al Tumor Cells Circulate in the Peripheral Blood of All Major Carcinomas but not in Healthy Subjects or Patients With Nonmalignant Diseases. 2005;10: 6897–6904. 10.1158/1078-0432.CCR-04-0378 15501967

[16] Baeuerle PA, Gires O. EpCAM (CD326) finding its role in cancer. Br J Cancer. 2007;96: 417–423. 10.1038/sj.bjc.6603494 17211480 PMC2360029

[17] Martowicz A, Seeber A, Untergasser G. The role of EpCAM in physiology and pathology of the epithelium. Histol Histopathol. 2016;31: 349–355. doi: 10.14670/HH-11-678 26493939 10.14670/HH-11-678

[18] Myung JH, Launiere CA, Eddington DT, Hong S. Enhanced tumor cell isolation by a biomimetic combination of e-selectin and anti-epcam: Implications for the effective separation of circulating tumor cells (CTCs). Langmuir. 2010;26: 8589–8596. 10.1021/la904678p 20155985 PMC2877147

[19] Went P, Vasei M, Bubendorf L, Terracciano L, Tornillo L, Riede U, et al Frequent high-level expression of the immunotherapeutic target Ep-CAM in colon, stomach, prostate and lung cancers. Br J Cancer. 2006;94: 128–135. 10.1038/sj.bjc.6602924 16404366 PMC2361083

[20] Winter MJ, Cirulli V, Briaire-de Bruijn IH, Litvinov S V. Cadherins are regulated by Ep-CAM via phosphaditylinositol-3 kinase. Mol Cell Biochem. 2007;302: 19–26. 10.1007/s11010-007-9420-y 17646933

[21] Winter MJ, Nagelkerken B, Mertens AEE, Rees-Bakker HAM, Briaire-de Bruijn IH, Litvinov S V. Expression of Ep-CAM shifts the state of cadherin-mediated adhesions from strong to weak. Exp Cell Res. 2003;285: 50–58. 10.1016/S0014-4827(02)00045-9 12681286

[22] Ladwein M, Pape UF, Schmidt DS, Schnölzer M, Fiedler S, Langbein L, et al The cell-cell adhesion molecule EpCAM interacts directly with the tight junction protein claudin-7. Exp Cell Res. 2005;309: 345–357. 10.1016/j.yexcr.2005.06.013 16054130

[23] Wu CJ, Feng X, Lu M, Morimura S, Udey MC. Matriptase-mediated cleavage of EpCAM destabilizes claudins and dysregulates intestinal epithelial homeostasis. J Clin Invest. 2017;127: 623–634. 10.1172/JCI88428 28094766 PMC5272188

[24] Wu CJ, Mannan P, Lu M, Udey MC. Epithelial cell adhesion molecule (EpCAM) regulates claudin dynamics and tight junctions. J Biol Chem. 2013;288: 12253–12268. 10.1074/jbc.M113.457499 23486470 PMC3636909

[25] Maghzal N, Vogt E, Reintsch W, Fraser JS, Fagotto F. The tumor-associated EpCAM regulates morphogenetic movements through intracellular signaling. J Cell Biol. 2010;191: 645–659. 10.1083/jcb.201004074 20974811 PMC3003323

[26] Pandya P, Orgaz JL, Sanz-Moreno V. Actomyosin contractility and collective migration: may the force be with you. Curr Opin Cell Biol. Elsevier Ltd; 2017;48: 87–96. 10.1016/j.ceb.2017.06.006 28715714 PMC6137077

[27] Sankpal N V., Fleming TP, Sharma PK, Wiedner HJ, Gillanders WE. A double-negative feedback loop between EpCAM and ERK contributes to the regulation of epithelial-mesenchymal transition in cancer. Oncogene. Nature Publishing Group; 2017;36: 3706–3717. 10.1038/onc.2016.504 28192403 PMC5571977

[28] Garcia MA, Nelson WJ, Chavez N. Cell–Cell Junctions Organize Structural and Signaling Networks. Cold Spring Harb Perspect Biol. 2017;10: 1–28. 10.1101/cshperspect.a029181 28600395 PMC5773398

[29] Gunzel D, Yu ASL. Claudins and the Modulation of Tight Junction Permeability. Physiol Rev. 2013;93: 525–569. 10.1152/physrev.00019.2012 23589827 PMC3768107

[30] Tanimura S, Chatani Y, Hoshino R, Sato M, Watanabe SI, Kataoka T, et al Activation of the 41/43 kDa mitogen-activated protein kinase signaling pathway is required for hepatocyte growth factor-induced cell scattering. Oncogene. 1998;17: 57–65. 10.1038/sj.onc.1201905 9671314

[31] Aoki K, Kondo Y, Naoki H, Hiratsuka T, Itoh RE, Matsuda M. Propagating Wave of ERK Activation Orients Collective Cell Migration. Dev Cell. Elsevier Inc.; 2017;43: 305–317.e5. 10.1016/j.devcel.2017.10.016 29112851

[32] Matsubayashi Y, Ebisuya M, Honjoh S, Nishida E. ERK Activation Propagates in Epithelial Cell Sheets and Regulates Their Migration during Wound Healing. Curr Biol. 2004;14: 731–735. 10.1016/j.cub.2004.03.060 15084290

[33] Nguyen DHD, Catling AD, Webb DJ, Sankovic M, Walker LA, Somlyo A V., et al Myosin light chain kinase functions downstream of Ras/ERK to promote migration of urokinase-type plasminogen activator-stimulated cells in an integrin-selective manner. J Cell Biol. 1999;146: 149–164. 10.1083/jcb.146.1.149 10402467 PMC2199739

[34] Tojkander S, Gateva G, Lappalainen P. Actin stress fibers—assembly, dynamics and biological roles. J Cell Sci. 2012;125: 1855–1864. 10.1242/jcs.098087 22544950

[35] Vicente-Manzanares M, Ma X, Adelstein RS, Horwitz AR. Non-muscle myosin II takes centre stage in cell adhesion and migration. Nat Rev Mol Cell Biol. 2009;10: 778–790. 10.1038/nrm2786 19851336 PMC2834236

[36] Reffay M, Parrini MC, Cochet-Escartin O, Ladoux B, Buguin A, Coscoy S, et al Interplay of RhoA and mechanical forces in collective cell migration driven by leader cells. Nat Cell Biol. 2014;16: 217–223. 10.1038/ncb2917 24561621

[37] Trepat X, Wasserman MR, Angelini TE, Millet E, Weitz DA, Butler JP, et al Physical forces during collective cell migration. Nat Phys. Nature Publishing Group; 2009;5: 426–430. 10.1038/nphys1269

[38] Gaber A, Kim SJ, Kaake RM, Benčina M, Krogan N, Šali A, et al EpCAM homo-oligomerization is not the basis for its role in cell-cell adhesion. Sci Rep. 2018;8: 13269 10.1038/s41598-018-31482-7 30185875 PMC6125409

[39] Dukes JD, Whitley P, Chalmers AD. The MDCK variety pack: Choosing the right strain. BMC Cell Biol. 2011;12: 2–5. 10.1186/1471-2121-12-2 21982418 PMC3209442

[40] Lu Z, Ding L, Hong H, Hoggard J, Lu Q, Chen YH. Claudin-7 inhibits human lung cancer cell migration and invasion through ERK/MAPK signaling pathway. Exp Cell Res. Elsevier Inc.; 2011;317: 1935–1946. 10.1016/j.yexcr.2011.05.019 21641901 PMC3134522

[41] Zhan T, Rindtorff N, Boutros M. Wnt signaling in cancer. Oncogene. Nature Publishing Group; 2017;36: 1461–1473. 10.1038/onc.2016.304 27617575 PMC5357762

[42] De Rooij J, Kerstens A, Danuser G, Schwartz MA, Waterman-Storer CM. Integrin-dependent actomyosin contraction regulates epithelial cell scattering. J Cell Biol. 2005;171: 153–164. 10.1083/jcb.200506152 16216928 PMC2171213

[43] Barth AIM, Pollack AL, Altschuler Y, Mostov KE, Nelson WJ. NH2-terminal deletion of β-catenin results in stable colocalization of mutant β-catenin with adenomatous polyposis coli protein and altered MDCK cell adhesion. J Cell Biol. 1997;136: 693–706. 10.1083/jcb.136.3.693 9024698 PMC2134296

[44] Bahmanyar S, Kaplan DD, DeLuca JG, Giddings TH, O’Toole ET, Winey M, et al β-catenin is a Nek2 substrate involved in centrosome separation. Genes Dev. 2008;22: 91–105. 10.1101/gad.1596308 18086858 PMC2151018

[45] Louie RK. Adenomatous polyposis coli and EB1 localize in close proximity of the mother centriole and EB1 is a functional component of centrosomes. J Cell Sci. 2004;117: 1117–1128. 10.1242/jcs.00939 14970257 PMC3368710

[46] Preibisch S, Saalfeld S, Tomancak P. Globally optimal stitching of tiled 3D microscopic image acquisitions. Bioinformatics. 2009;25: 1463–1465. 10.1093/bioinformatics/btp184 19346324 PMC2682522

