# Supplementary figures and images for: Regulation of epithelial migration by epithelial cell adhesion molecule requires its Claudin-7 interaction domain
Source: PLoS One. 2018 Oct 10;13(10):e0204957. doi: 10.1371/journal.pone.0204957 (PMC6179577; doi:10.1371/journal.pone.0204957)

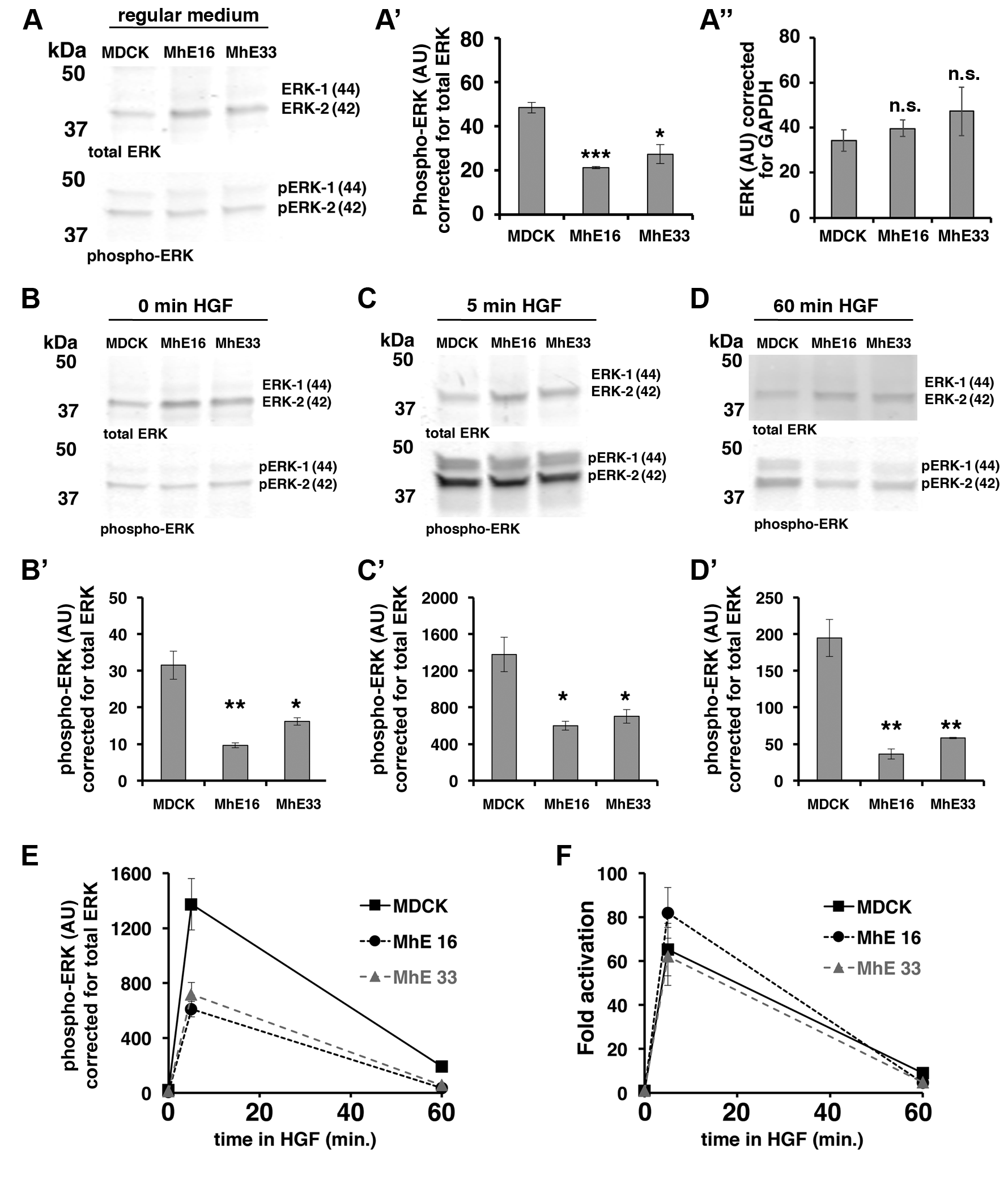

Supplement: S1 Fig — (A) Wildtype MDCK, and MDCK lines overexpressing human EpCAM (MhE16, MhE33) were SDS extracted one day after plating at subconfluent cell density; levels of ERK and phospho-ERK were analyzed in the same immunoblot. (A’) The graph shows quantification of combined 44 and 42 kDa phospho-ERK protein levels normalized to combined 44 and 42 kDa ERK protein levels in the same sample. Error bars: S.E.M. of three independent samples for each cell line; p values derived from unpaired Student’s t test: *** p = 0.0004 for MhE16 to MDCK and * p = 0.0128 for MhE33 to MDCK. (A”) The graph shows quantification of combined 44 and 42 kDa total ERK protein levels normalized to GAPDH protein levels in the same sample. Error bars: S.E.M. of three independent samples for each cell line; total ERK levels are not significantly different in these three cell lines. (B-D) MDCK cells and MDCK cell lines MhE16, MhE33 overexpressing human EpCAM were plated at low density for one day, serum-starved for 2 hours and extracted (B), or serum-starved for 2 hours and treated with 5 ng/ml HGF for 5 minutes (C) or 60 minutes (D). Cells were SDS extracted and levels of ERK and phospho-ERK were analyzed in the same immunoblot. (B’-D’) The graphs show quantification of combined 44 and 42 kDa phospho-ERK protein levels normalized to combined 44 and 42 kDa ERK protein levels in the same sample. Arbitrary units for protein intensities in Y-axis (AU) x103; error bars: S.E.M. of three independent samples for each cell line; *, **p values compared to MDCK cells derived from unpaired Student’s t test. In (B’) MhE16 **p = 0.0049, MhE33 *p = 0.0179; in (C’) MhE16 *p = 0.0161, MhE33 *p = 0.0288; in (D’) MhE16 **p = 0.0038, MhE33 **p = 0.0057. (E) Phospho-ERK levels from graphs of serum-starved (0) cells in (B’), or cells treated 5 minutes (C’) or 60 minutes (D’) with HGF are combined into one graph in (E) to compare HGF-induced phospho-ERK activation over time in these cell lines. Arbitrary units for protein intensit [file pone.0204957.s001.tif]

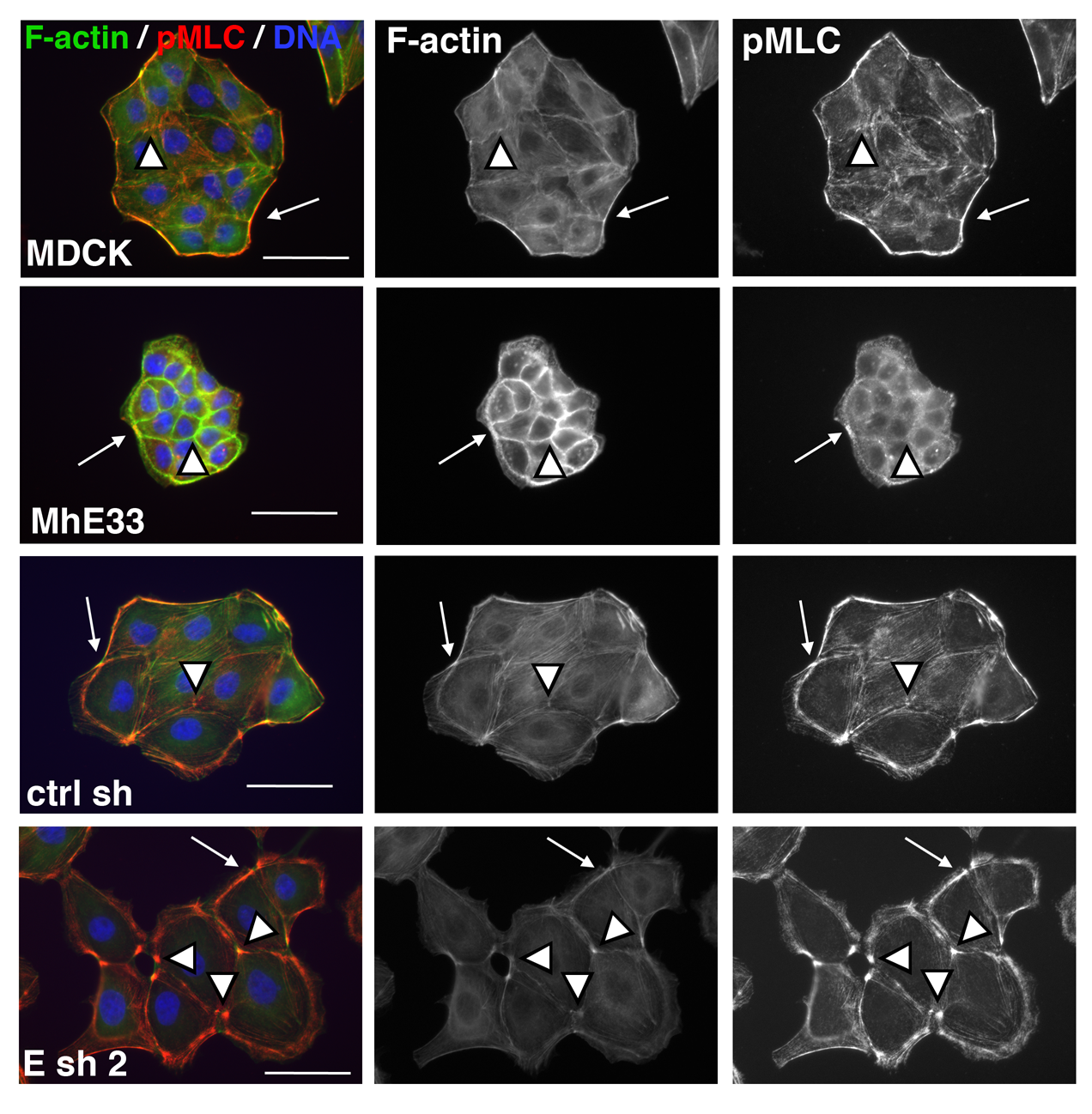

Supplement: S2 Fig — (A) Examples of smaller colonies of cells cultured and images as described in Fig 4A. Phospho-myosin-rich areas of cortical F-actin at the edge of colonies are marked with arrows and phospho-myosin-rich multicellular junctions inside colonies are marked with arrowheads. Bars = 50μm. (TIF) [file pone.0204957.s002.tif]

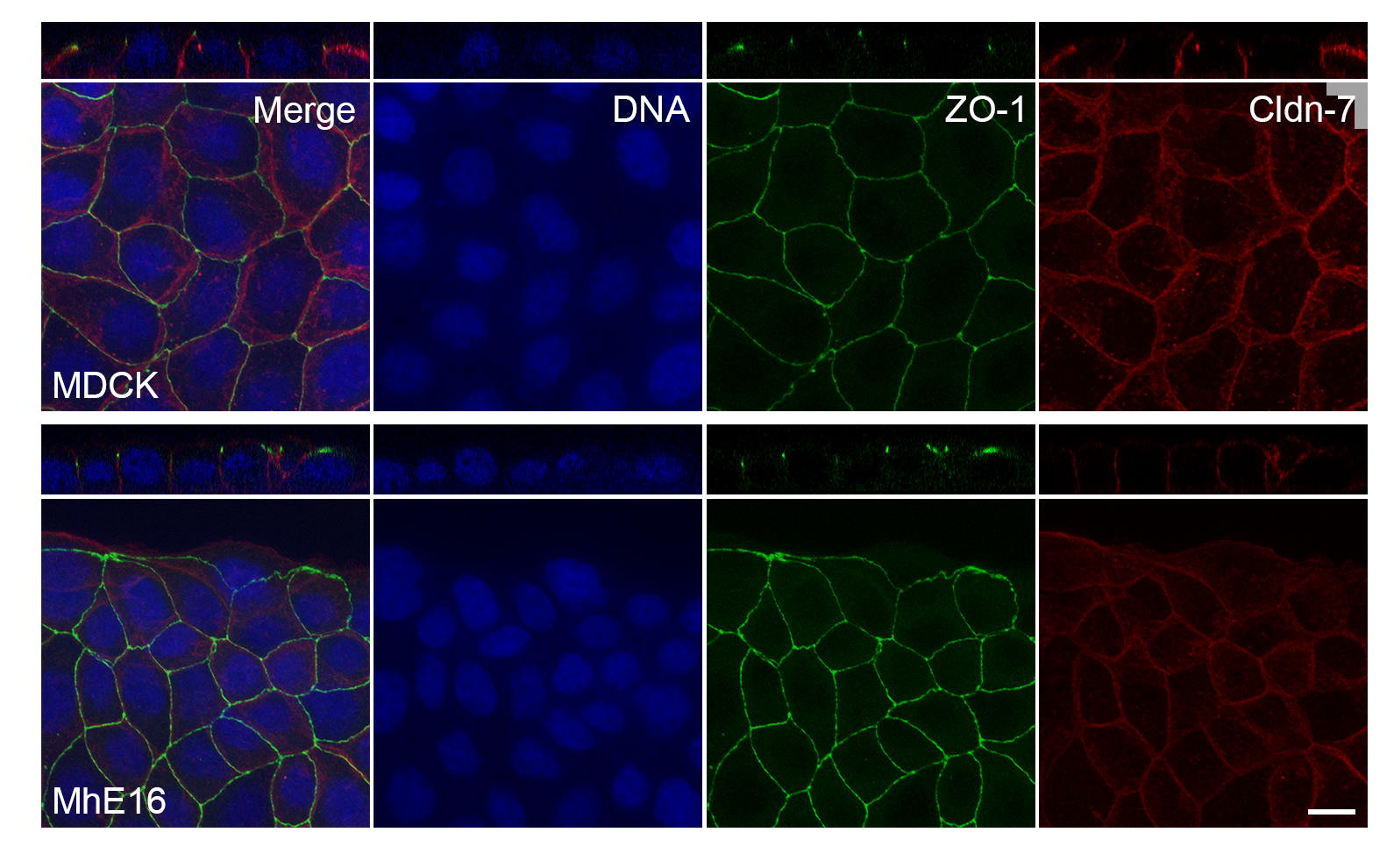

Supplement: S3 Fig — Confocal images of cells prepared as in Fig 5C, stained for nuclei (blue) tight-junction marker ZO-1 (green) and Claudin-7 (red). In both MDCK and MhE16 lines, Claudin-7 localizes along the entired basolateral membrane, whereas ZO-1 is restricted to the apical side of the lateral membrane corresponding to the tight junctions. Scale bar is 10μm. (TIF) [file pone.0204957.s003.tif]

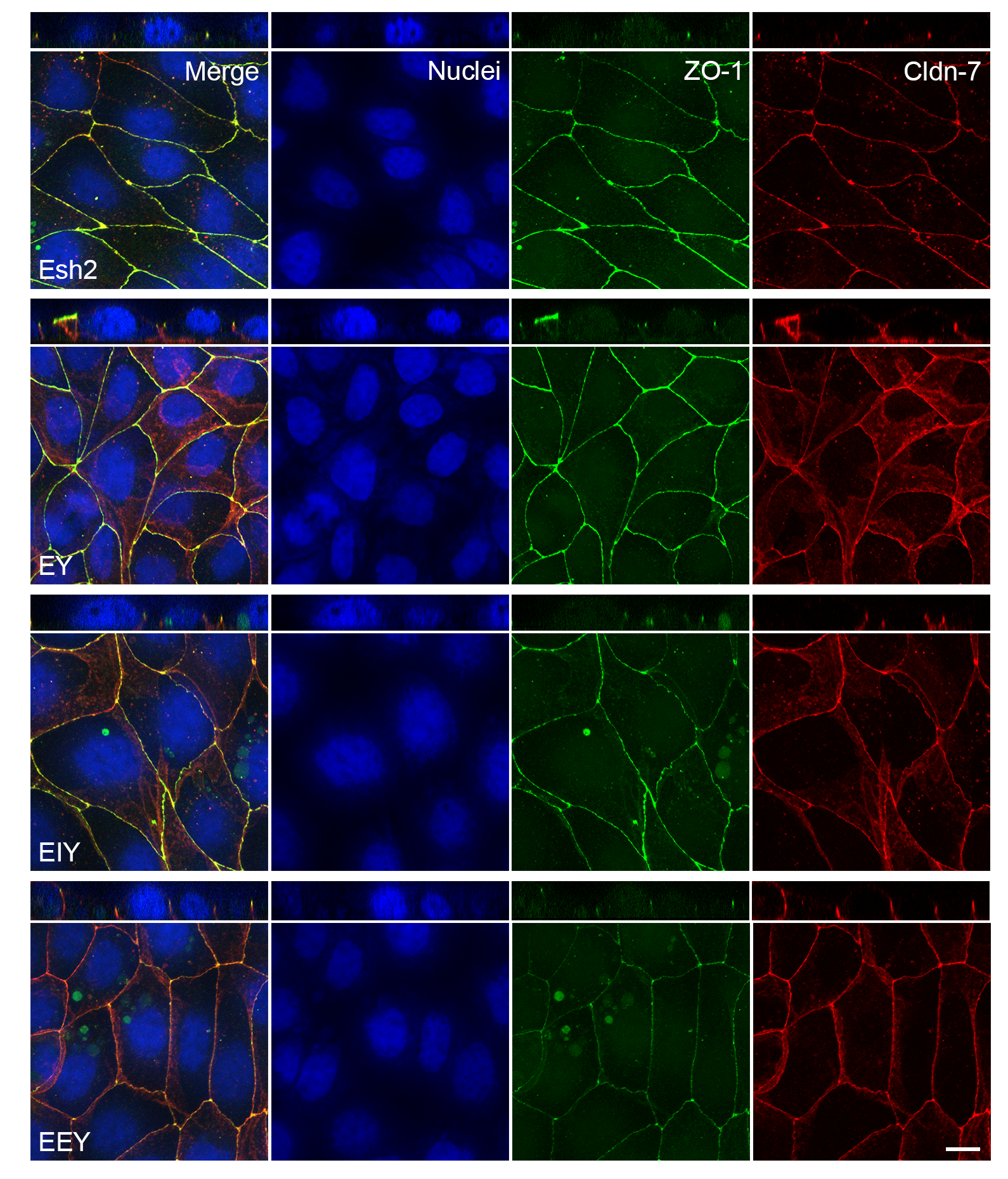

Supplement: S4 Fig — Confocal images of cells prepared as in Fig 5C, stained for nuclei (blue) tight-junction marker ZO-1 (green) and Claudin-7 (red). In the Esh2 line, Claudin-7 colocalizes with the ZO-1 and is restricted to the apical side of the lateral membrane corresponding to the tight junctions. In the EY, EIY and EEY lines the Claudin-7 localization is rescued and once again distributes along the basolateral membrane, while ZO-1 remains restricted to the tight junctions. Scale bar is 10μm. (TIF) [file pone.0204957.s004.tif]

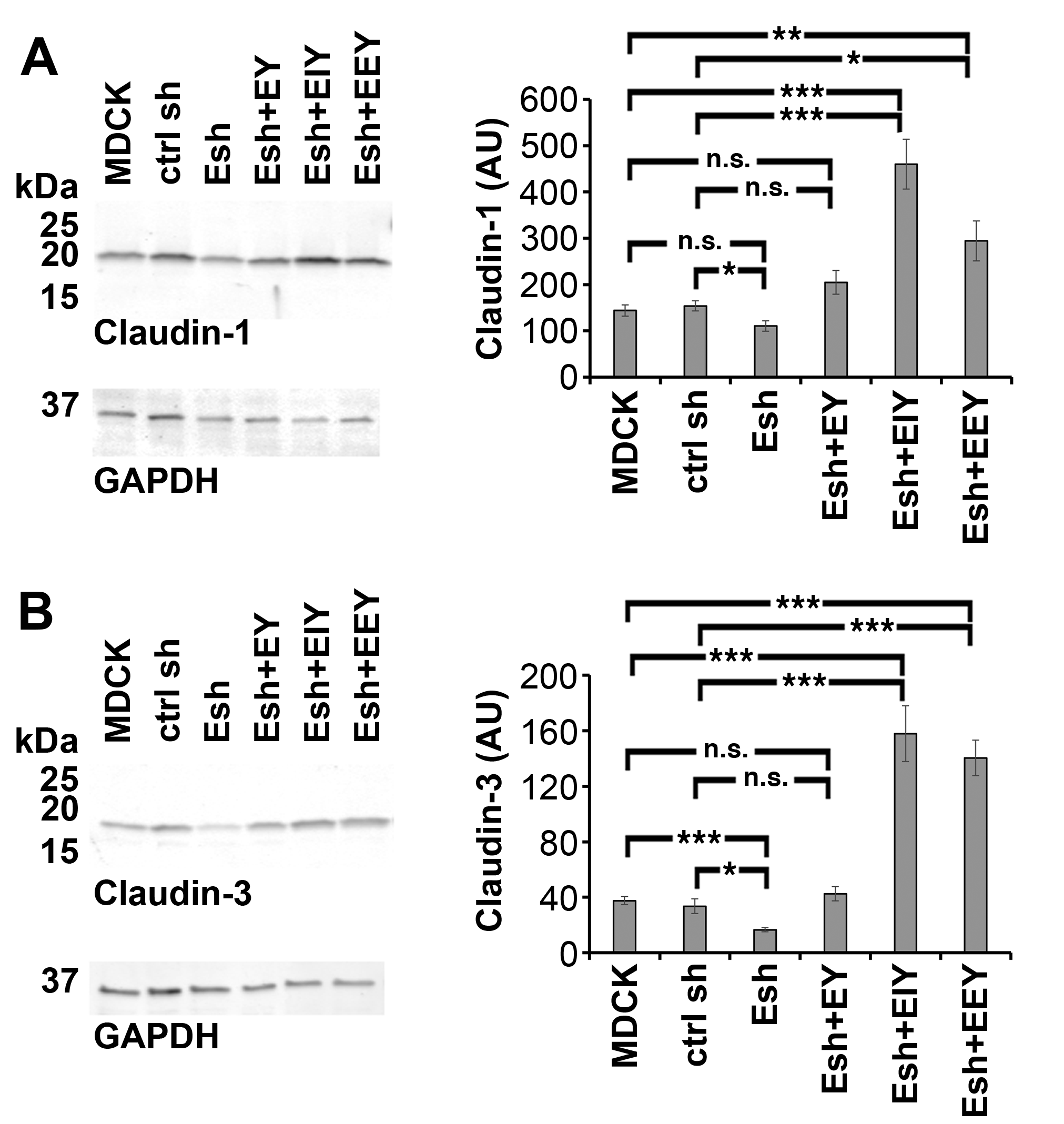

Supplement: S5 Fig — (A) Claudin-1 and (B) Claudin-3 protein levels were analyzed as described for Claudin-7 in Fig 6. Graphs show quantifications of indicated proteins normalized to GAPDH levels in the same sample. Arbitrary units for protein intensities in Y-axis (AU) x103; error bars: S.E.M. of six samples for each cell line. Protein extracts are the same as in Fig 6. (A) Expression of Claudin-1 is only slightly reduced, (B) expression of Claudin-3 is more strongly reduced in EpCAM-depleted Esh2 cells (Esh) compared to MDCK and control shRNA line (ctrl sh). Expression of EY, EIY or EEY in Esh2 rescues Claudin-1 and -3 protein levels in these cell lines compared to levels in the Esh line. Higher EpCAM and Claudin-7 levels in the EIY and EEY lines compared to parental MDCK or ctrl sh control lines (see Fig 6). (TIF) [file pone.0204957.s005.tif]

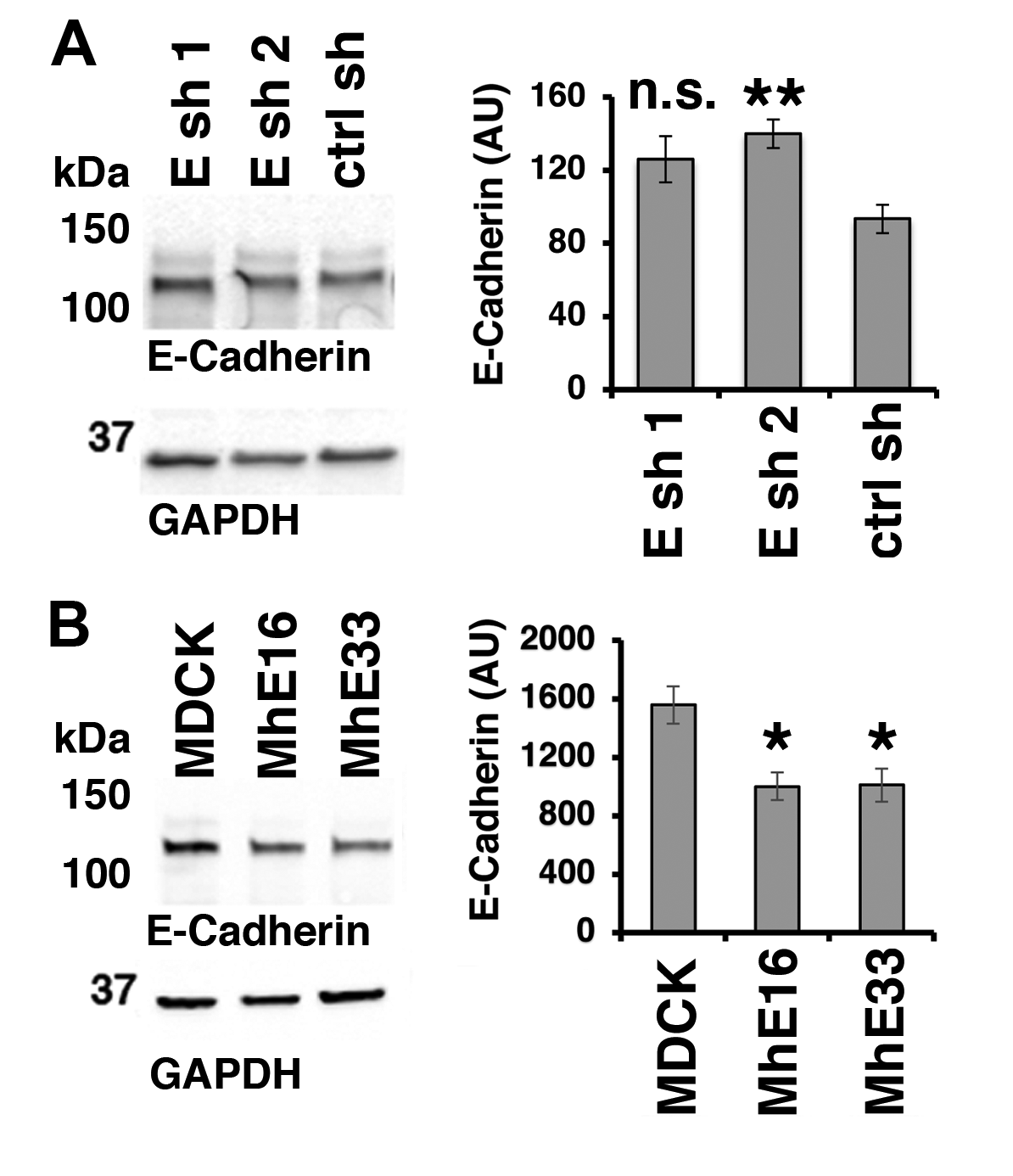

Supplement: S6 Fig — The same protein extracts as shown in Fig 5A and 5B, respectively, were immunoblotted for E-Cadherin and E-cadherin levels were normalized for GAPDH. Arbitrary units for protein intensities in Y-axis (AU) x103; error bars: S.E.M. of four samples for each cell line in (A) and three samples for each cell line in (B); p values derived from unpaired Student’s t test: ** p = 0.0055 for Esh2 to ctrl sh; * p = 0.025 for MhE16 to MDCK and * p = 0.016 for MhE33 to MDCK. EpCAM-depleted Esh2 MDCK cells have slightly more E-cadherin (A) whereas EpCAM-overexpressing MDCK cells have slightly less E-cadherin (B). (TIF) [file pone.0204957.s006.tif]
